# Global spatiotemporal trends and influencing factors of type 2 diabetes mellitus mortality: estimates and predictions from 1990 to 2040

**DOI:** 10.3389/fendo.2025.1601089

**Published:** 2025-08-28

**Authors:** Yue Zhang, Huan Deng, Jian Zu, Yujiao Deng, Jingyue Tan, Zhanpeng Yang, Yang Jiao, Xiaomeng Cui, Lei Zhang, Fanpu Ji, Yuan Wang

**Affiliations:** ^1^ School of Mathematics and Statistics, Xi’an Jiaotong University, Xi’an, China; ^2^ National & Local Joint Engineering Research Center of Biodiagnosis and Biotherapy, the Second Affiliated Hospital of Xi’an Jiaotong University, Xi’an, China; ^3^ Division of Gastroenterology, the Second Affiliated Hospital of Xi’an Jiaotong University, Xi’an, China; ^4^ Department of Endocrinology, the Second Affiliated Hospital of Xi’an Jiaotong University, Xi’an, China; ^5^ Department of Infectious Diseases, the Second Affiliated Hospital of Xi’an Jiaotong University, Xi’an, China; ^6^ China-Australia Joint Research Centre for Infectious Diseases, School of Public Health, Xi’an Jiaotong University Health Science Centre, Xi’an, China; ^7^ Artificial Intelligence and Modelling in Epidemiology Program, Melbourne Sexual Health Centre, Alfred Health, Melbourne, VIC, Australia; ^8^ Central Clinical School, Faculty of Medicine, Monash University, Melbourne, VIC, Australia; ^9^ Key Laboratory of Environment and Genes Related to Diseases, Xi’an, Jiaotong University, Ministry of Education of China, Xi’an, China; ^10^ Key Laboratory of Surgical Critical Care and Life Support (Xi’an Jiaotong University), Ministry of Education, Xi’an, China; ^11^ Global Health Institute, School of Public Health, Xi’an Jiaotong University Health Science Center, Xi’an, China

**Keywords:** type 2 diabetes mellitus, age-standardized mortality rate, decomposition analysis, frontier analysis, Bayesian spatio-temporal model

## Abstract

**Background:**

To predict global spatiotemporal trends and influencing factors of type 2 diabetes mellitus (T2DM) mortality.

**Materials and methods:**

Based on the Global Burden of Disease 2021 database, we utilized the Bayesian Age-Period-Cohort model to predict the age-standardized mortality rate (ASMR) of T2DM in 2022–2040. The common spatial pattern and risk factors were forecasted using the Bayesian spatiotemporal model. Decomposition analysis determined the contribution of influencing factors to T2DM deaths. Frontier analysis estimated the gaps between countries and the potential to reduce ASMR.

**Results:**

The global deaths of T2DM have been growing, with ASMR showing an increasing and then gradually decreasing trend in 1990–2040 (AAPC_1990–2021_, 0.31 [0.21–0.42]; AAPC_2022–2040_, −0.16 [−0.17 to −0.15]), and they are projected to reach 2,756,631 and 18.63 (8.82–28.43) per 100,000 person by 2040, respectively. The ASMR of males is always higher than that of females except Eastern Mediterranean and increases fastest in the 15–49-year group. South-East Asia will have the largest number of deaths (1,035,666 in 2040) and the fastest growth (AAPC_2022–2040_, 0.22 [0.216–0.228]) in 2022–2040, but Africa will always have the highest ASMR. Age structure contributes more than 58.80% to the increase in deaths except Africa. There are 72 countries with a higher ASMR in 2040 compared to 2021, and 86 countries will have a heavier burden in 2022–2040. High-middle SDI countries have a higher ASMR with huge potential to reduce them.

**Conclusions:**

The global deaths of T2DM have been growing, Africa will always have the highest ASMR. Age structure will be the main factor influencing T2DM mortality across regions.

## 1 Introduction

Diabetes mellitus is the fastest-growing health emergency of the 21st century. According to the World Health Statistics Report for 2023, in 2019 alone, diabetes mellitus caused the deaths of 2 million people worldwide, with type 2 diabetes mellitus (T2DM) being predominant (1). Currently, there are more than 500 million patients with diabetes mellitus globally, which is projected to reach 1.3 billion by 2050 (2). According to the IDF Diabetes Atlas report, in 2021, global healthcare expenditure for diabetes mellitus amounted to a staggering $966 billion, accounting for 9% of total global health expenditure (3). More than 80% of T2DM patients reside in low- and middle-income countries (4). The burden of this disease remains high, posing major challenges for individuals, families, and societies (5).

The burden of T2DM is primarily determined by risk factors, with varying contributions according to region (6–8). Obesity, high fasting glucose, unhealthy lifestyle, dietary habits, air pollution, and smoking are all major risk factors driving the increase in T2DM (9). Dysfunctions in sugar and lipid metabolism affect vascular integrity and supply, resulting in macrovascular and microvascular disorders, organ dysfunction, and premature death (4). Patients with T2DM have a two- to fourfold higher risk of death and cardiovascular events than the general population (10). The right care and services can go a long way in preventing T2DM and death. Collectively, the manifestations of diabetes mellitus result in enormous human suffering and huge economic costs. Studying the risk factors and disease burden of diabetes mellitus mortality is important for optimizing the allocation of medical resources. This study aims to evaluate the spatiotemporal changes in age-standardized mortality rates (ASMR) of T2DM across 204 countries and regions from 1990 to 2040, and to explore the impacts of different influencing factors on the burden of ASMR and deaths.

## 2 Materials and methods

### 2.1 Study design, setting, and population

We obtained ASMR per 100,000 population from Global Burden of Disease (GBD) 2021 for six World Health Organization (WHO) regions and 204 countries and territories globally from 1990 to 2021,disaggregated by sex and age groups (15–49 years, 50–74 years, and ≥75 years). The GBD database covers epidemiological data for 369 diseases and 87 attributable risk factors in all member countries of the WHO. GBD used the Cause of Death Ensemble model (CODEm) to predict diabetes mellitus mortality rates (11).

### 2.2 Definition and data sources

The projected demographic data of the six WHO regions were from the population module World Population Prospects 2022 (https://population.un.org/wpp/) of the United Nation official website. We downloaded the exposure value of high fasting plasma glucose (HFPG) from GBD 2021. GBD defines HFPG as fasting blood glucose ≥7 mmol/L. We also obtained sociodemographic index (SDI) from GBD 2021, which was used to measure the level of social and economic development of a country or region. Segi’s world standard population was used to calculate the age-standardized rates in GBD 2021.

### 2.3 Statistical analyses

We used the Bayesian Age-Period-Cohort (BAPC) model to predict the ASMR for different regions and countries as well as deaths by region in 2022–2040. The average annual percent change (AAPC) in ASMR during 1990–2021 and 2022–2040 was measured using the Joinpoint regression model. The Bayesian spatiotemporal model characterized the common spatiotemporal trends of ASMR across countries during 1990–2040.

Decomposition analysis was used to determine the contribution of age structure, population growth, and epidemiological variations to the change of deaths. We also quantified the impact of SDI and HFPG on ASMR by region using the Bayesian spatiotemporal model, where the HFPG and SDI for 2022–2040 per country were predicted by the Auto-ARIMA and Prophet models. Frontier analysis utilized the SDI as a measure of development level for countries to assess the ASMR in 2021 and 2040. Moreover, countries with a similar SDI were compared to get the gap between them and find the lowest achievable AMSR for them. The uncertainty intervals (UI) were calculated by Markov chain Monte Carlo (MCMC). Further details regarding the models can be found in the **Supplementary Materials**. All statistical analyses were conducted using R (version 4.4.0).

## 3 Results

### 3.1 The overall deaths and ASMR for type 2 diabetes mellitus, 1990–2040

**Figure 1:**
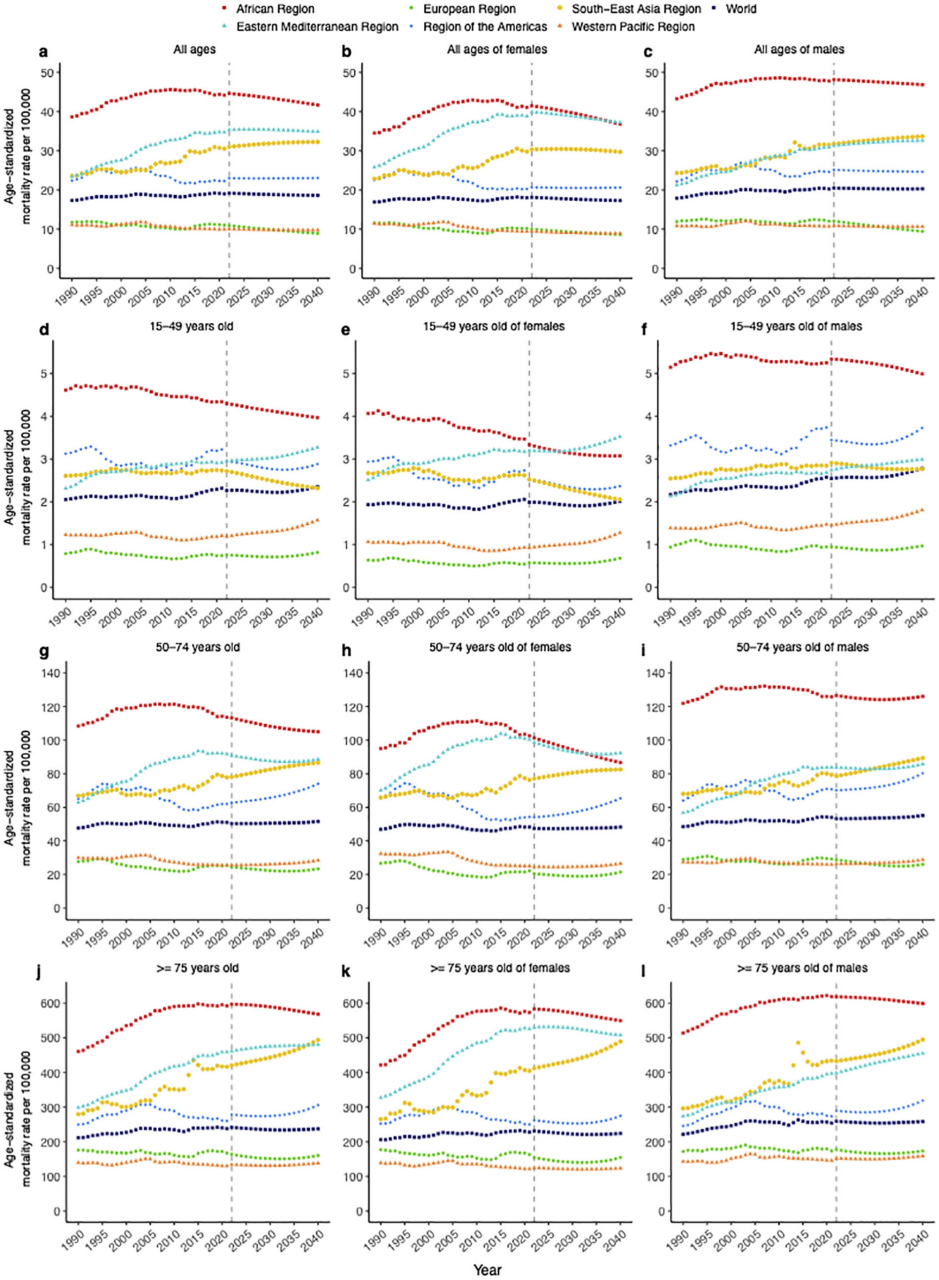
Temporal trends of T2DM age-standardized mortality rates in 1990–2040, stratified by sex, age, and region. **(a)** All ages, **(b)** all ages of females, **(c)** all ages of males, **(d)** 15–49 years old, **(e)** 15–49 years old of females, **(f)** 15–49 years old of males, **(g)** 50–74 years old, **(h)** 50–74 years old of females, **(i)** 50–74 years old of males, **(j)** ≥75 years old, **(k)** ≥75 years old of females, **(l)** ≥75 years old of males.

The global number of deaths due to T2DM has been rising from 632,322 (95% UI, 596,870–662,082) in 1990 to 1,608,123 (1,493,438–1,708,294) in 2021 and is projected to be 2,756,631 in 2040 (**Supplementary Table S1**). However, the ASMR increased from 17.33 (16.20–18.18) per 100,000 person in 1990 to 19.02 (17.57–20.20) in 2021 and is projected to decrease to 18.63 (8.82–28.43) in 2040 (**Supplementary Table S2**). The AAPC values of ASMR are 0.31 (0.21–0.42) in 1990–2021 and −0.16 (−0.17 to −0.15) in 2022–2040, which also show an increasing and then gradually decreasing trend over 1990–2040 (**Figure 1a**). The ASMR of men is always higher than that of women (20.27 vs. 18.03 in 2021, 20.30 vs.17.29 in 2040) and has an AAPC of 0.45 (0.32–0.58) twice as high as that of women in 1990–2021. It will remain stable for men (AAPC_2022–2040_, −0.05 [−0.07 to −0.04]) but slowly decline for women (AAPC_2022–2040_, −0.26 [−0.27 to −0.24]) in 2022–2040 (**Figures 1b, c**). In terms of age, the ≥75-year group has the largest ASMR and its ASMR is more than 100 times higher than those in the 15–49-year group in 2021 (237.96 vs. 2.32), projected to reach 237.48 (133.44–389.07) by 2040. However, the 15–49-year group for males is still a concern (AAPC_1990–2021_, 0.57 [0.46–0.67]; AAPC_2022–2040_, 0.48 [0.44–0.52]), which is the fastest growing compared to other age groups (**Figures 1d–l**).

### 3.2 The deaths and ASMR for type 2 diabetes mellitus by region, 1990–2040

Regionally, the number of deaths increases in all six regions throughout the period 1990–2040. South-East Asia has the largest number of deaths and gradually increases, projected to rise from 491,202 (445,841–540,161) in 2021 to 1,035,666 in 2040 (37.57% of global deaths, **Supplementary Table S1**), while the Eastern Mediterranean remains at a lower level. As for ASMR, **Figure 1a** indicates that Africa, South-East Asia, the Americas, and the Eastern Mediterranean regions have higher ASMRs than the global in the period 1990–2040, and Africa consistently has the highest ASMR reaching 41.68 (25.65–57.72) per 100,000 persons in 2040. The fastest growth of ASMR in 1990–2021 is in Eastern Mediterranean (AAPC_1990–2021_, 1.30 [1.19–1.41]) reaching 34.87 (15.00–54.73) until 2021, but in 2022–2040 it is in South-East Asia (AAPC_2022–2040_, 0.22 [0.216–0.228]) reaching 32.26 (9.78–54.74) by 2040 (**Supplementary Table S2**). Males have a significantly higher ASMR than females in the Africa and the Americas, with the same phenomenon in all three age groups. Notably, there is an opposite situation in the Eastern Mediterranean region, where the female ASMR is 1.20 times higher than the male ASMR (**Figures 1b, c**). Regardless of region and gender, the ≥75-year group has the largest ASMR, projected to remain the largest at 568.50 (386.57–808.57) in Africa by 2040, and except for a slight decline in Africa and Europe, all regions will continue to rise in the future especially South-East Asia. The 15–49- and 50–74-year groups have the fastest growth rates for both sexes in the Eastern Mediterranean until 2021 and are projected to have the largest ASMRs for females in this region in 2040. However, they will grow fastest in the Western Pacific and the Americas during 2022–2040, respectively (**Figures 1d–l**).

### 3.3 The influencing factors for type 2 diabetes mellitus mortality by region

**Figure 2:**
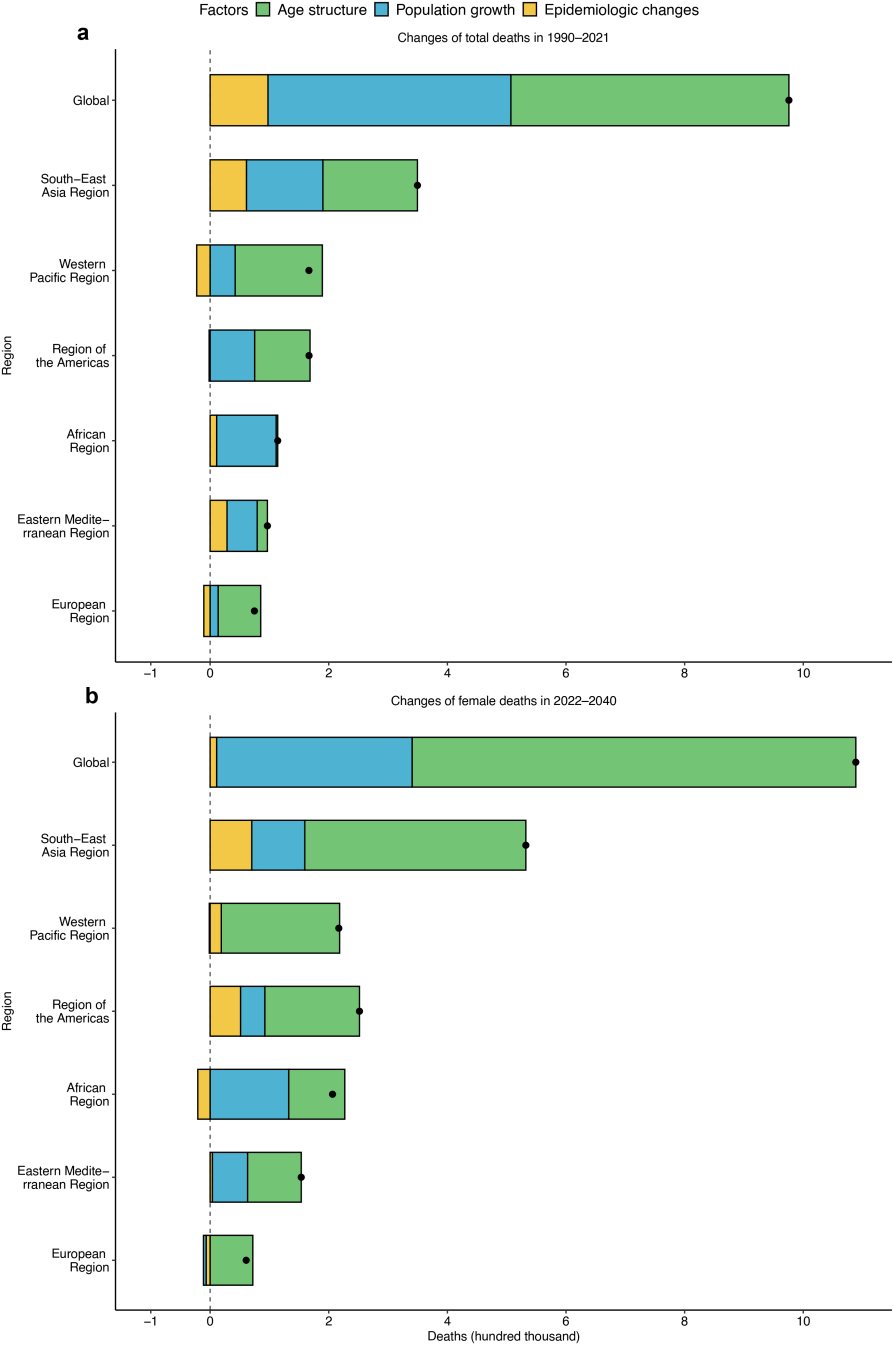
Contributions of population growth, age structure, and epidemiological changes to the increase in T2DM deaths globally and in six regions. **(a)** 1990–2021 and **(b)** 2022–2040. Black dots represent the growth in deaths during the two time periods. The magnitude of a positive value indicates a corresponding increase in deaths attributed to the component, and the magnitude of a negative value indicates a corresponding decrease in deaths attributed to the related component.

Based on T2DM deaths, we evaluate the effects of age structure, population growth, and epidemiological changes. Age structure (48.04%) and population growth (41.96%) were the main factors leading to the increase in global deaths from 1990 to 2021. Population growth contributed the most to deaths in Africa (88.04%) and Eastern Mediterranean (52.64%), while the largest contribution was from age structure in other regions (**Figure 2a**, **Supplementary Table S3**). Except Africa, the contribution of age structure is projected to increase, remaining the largest contributor and more than 58.80% to the increase in deaths globally and elsewhere in 2022–2040. Epidemiologic changes will lead to an increase (8.67%) in deaths in the Western Pacific region but a 10.05% decrease in Africa, which means that the mortality situation in Africa will be improved (**Figure 2b**). In general, except Africa, the change in age structure is still the most important cause of increase in deaths.

Furthermore, we explore the effects of HFPG and SDI (**Supplementary Table S4**, **Supplementary Table S5**) in ASMR. Over 1990–2021, ASMR is positively associated with HFPG and negatively associated with SDI, and the effect of HFPG is greater than that of SDI in ASMR. The global ASMR increased by 20.88 (19.52–22.25) for each unit increase in HFPG, while it decreased by 18.27 (19.26–17.28) for each unit increase in SDI. The effects of HFPG and SDI in ASMR were most pronounced in the Western Pacific 23.20 (20.60–25.79) and the Americas −25.04 (−28.22 to −21.85), respectively. Based on the results from 1990 to 2040, it can be estimated that their impact will be reduced except for the impact of HFPG on Europe in 2022–2040, and Europe must seek measures to control this situation (**Table 1**).

**Table 1:** Impact of high fasting plasma glucose and sociodemographic index in type 2 diabetes mellitus mortality.

Region	Countries	High fasting plasma glucose		Sociodemographic index		Interaction term	
		1990–2021	1990–2040	1990–2021	1990–2040	1990–2021	1990–2040
Global	202	20.88 (19.52–22.25)	13.31 (12.34–14.28)	−18.27 (−19.26 to −17.28)	−12.53 (−13.35 to −11.70)	−7.69 (−8.27 to −7.10)	−5.85 (−6.24 to −5.47)
Africa	47	17.53 (15.04–20.03)	9.05 (7.31–10.79)	−4.79 (−7.42 to −2.18)	−1.86 (−3.68 to −0.04)	1.94 (0.51–3.37)	−1.55 (−2.47 to −0.64)
Eastern Mediterranean	21	5.77 (3.23–8.31)	1.61 (-0.44–3.66)	−2.68 (−5.52–0.15)	4.72 (2.50–6.94)	−3.39 (−4.60 to −2.19)	−6.20 (−7.04 to −5.37)
Europe	55	21.43 (18.45–24.42)	27.37 (25.17–29.58)	−24.36 (−25.74 to −22.99)	−22.30 (−23.52 to −21.09)	−11.56 (−13.86 to −9.26)	−10.35 (−11.82 to −8.88)
Americas	39	20.82 (17.31–24.34)	3.00 (0.38–5.61)	−25.04 (−28.22 to −21.85)	−4.71 (−6.97 to −2.44)	−15.76 (−17.40 to −14.12)	−4.54 (−5.60 to −3.48)
South-East Asia	11	16.67 (10.03–23.32)	16.02 (11.82–20.24)	−9.42 (−14.28 to −4.56)	−1.87 (−4.93–1.18)	1.76 (−2.77–6.29)	2.61 (0.14–5.09)
Western Pacific	29	23.20 (20.60–25.79)	17.09 (15.01–19.17)	−15.31 (−18.18 to −12.44)	−3.73 (−5.77 to −1.70)	−6.21 (−7.37 to −5.05)	−6.34 (−7.15 to −5.53)

Coefficients are posterior means (95% uncertainty intervals) from the Bayesian model. Negative/positive values indicate protective/risk effects, respectively.

### 3.4 The ASMR for type 2 diabetes mellitus by country, 1990–2040

**Figure 3:**
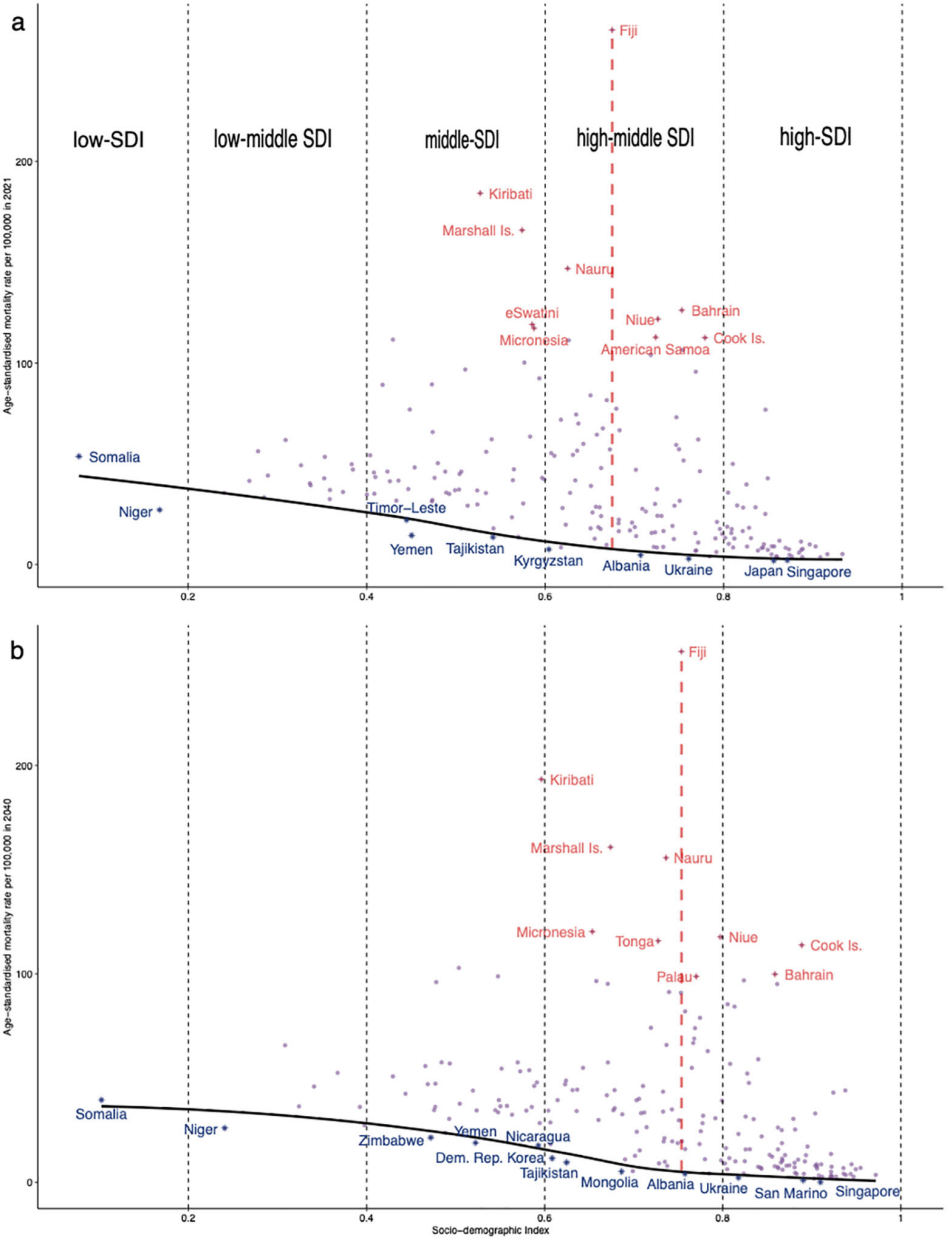
Frontier analysis based on sociodemographic index and age-standardized mortality rate for T2DM in **(a)** 2021 and **(b)** 2040. The black solid line represents the frontier line (optimal age-standardized mortality rate corresponding to each sociodemographic index), light purple dots represent the (predicted) ASMR for each country in 2021 or 2040, dark-blue dots represent countries where the ASMR are near the front line, and red dots represent the top 10 countries with the greatest effective differences.

ASMR has significant variation across the world in 2021 and 2040. Countries in central and southern Africa, South-East Asia, and Central America have high ASMRs, contrasting with Canada, China, Japan, and some European countries (**Supplementary Figure S1**). There are 72 (35.29%) countries with a higher ASMR in 2040, 13 of which have increases over 10, with the largest increase in Saint Lucia (57.11 vs. 91.30, **Supplementary Table S6**). By SDI, middle and high-middle SDI countries have a large ASMR; there are huge gaps between them for middle SDI countries in 2021 and for high-middle SDI countries in 2040, such as Fiji and Albania (265.22 vs. 4.67; 254.72 vs. 4.09), suggesting that there is greater potential to reduce ASMR in these countries (e.g., Fiji can reduce to 8.15 in 2021 and 5.62 in 2040) if they make full use of the SDI (**Figures 3a, b**, **Supplementary Table S7**). Furthermore, 12 countries including Singapore will be the top performers in their SDI areas in 2040, but Timor-Leste, Kyrgyzstan, and Japan among the top 10 performers in 2021 will need to work hard in 2040.

**Figure 4:**
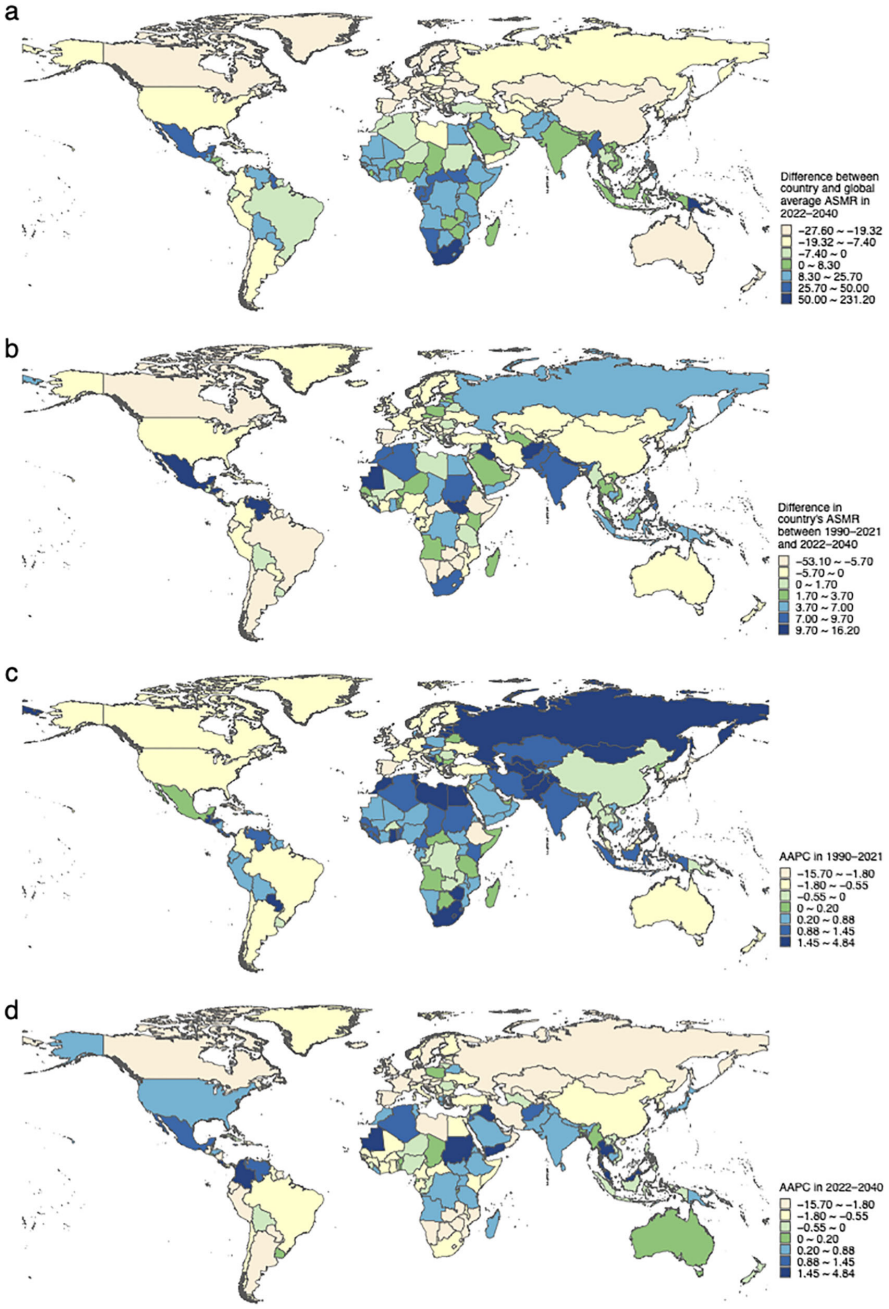
The common spatial pattern and the AAPC of T2DM age-standardized mortality rates across countries. **(a)** The common spatial pattern represents the difference in ASMR of the country compared to the global average level throughout 2022–2040. **(b)** The difference in a country’s ASMR between the periods 1990–2021 and 2022–2040. **(c)** The AAPC in 1990–2021. **(d)** The AAPC in 2022–2040.

The common spatial distributions over the entire periods 1990–2021 and 2022–2040 are also different; the global average levels of 1990–2021 and 2022–2040 are 31.03 (29.37–32.71) and 28.60 (26.88–30.33), respectively. In 1990–2021, island countries such as Fiji (255.13) and Kiribati (172.03) had the highest ASMR, while countries such as Japan (3.31) and Ukraine (3.37) had very low ASMR. During 2022–2040, island countries and South Africa (79.15) showing dark blue in **Figure 4a** will keep the higher ASMR, while San Marino (1.02) and Singapore (1.32) will have the lowest ASMR. Moreover, 86 (42.16%) countries globally will have a heavier mortality burden of T2DM in 2022–2040 by about 1.2 times as much as in 1990–2021 (**Supplementary Table S6**). They are mainly located in northern Africa and South-East Asia, with the greatest increase occurring in Kiribati (16.17) (**Figure 4b**). T2DM mortality in these countries will worsen in the future or the effects of the measures implemented will not be strong enough.

The growth trends of ASMR in 1990–2021 and 2022–2040 are markedly different. In 1990–2021, 126 (61.76%) countries showed an increase in ASMR with Russia growing the fastest (AAPC_1990–2021_, 4.8 [3.17–6.53]) (**Figure 4c**, **Supplementary Table S6**). Still, 66 (32.35%) countries show a growing trend in ASMR and the top five fastest-growing countries are Malta (AAPC_2022–2040_, 3.50 [3.41–3.58]), Costa Rica 3.18 (2.95–3.42), Saint Lucia 2.66 (2.62–2.70), Malaysia 2.61 (2.59–2.64), and N. Mariana Is. 2.36 (2.33–2.38) in 2022–2040 (**Supplementary Table S6**). Meanwhile, 22 countries show completely opposite ASMR trends in the two periods and are growing in 2022–2040, with Malta, Saint Lucia, Japan (AAPC_2022–2040_, 0.29 [0.26–0.33]), Colombia 2.229 (2.228–2.23), and Malaysia in particular requiring special attention (**Figure 4d**, **Supplementary Table S6**). These countries must give high priority to the situation and explore the reasons for the resurgence of ASMR in T2DM.

## 4 Discussion

This study conducted an in-depth analysis of the temporal trends, spatial patterns, and impact of influencing factors on deaths and ASMR of T2DM in 204 countries and regions globally from 1990 to 2040. The global number of deaths due to T2DM shows an increasing trend over 1990–2040. The ASMR for T2DM shows an increasing and then gradually decreasing trend over 1990–2040. The mortality burden is higher in males than in females, with the highest mortality rate observed in the elderly population. The burden of mortality is increasing most rapidly among young males. Africa consistently had the highest ASMR over 1990–2040. The South-East Asian region has both the highest mortality number of deaths and the fastest increasing trend in ASMR in 2022–2040. The increase in deaths is mainly driven by age structure. Additionally, HFPG and SDI are influential factors affecting mortality rates, particularly evident in the Western Pacific and the Americas. In fact, over the past 20 years, the global ASMR of T2DM has remained at a relatively high level, with a more significant increase observed in males (5). Studies have also shown that diabetes mellitus has become one of the main causes of disability worldwide for people aged 50 and above (12). These are consistent with our findings. Although the global mortality burden showed an upward trend from 1990 to 2021, it is expected to decline from 2022 to 2040, which may be attributed to advancements in medical technology, early intervention for diabetes mellitus, and strengthened health education. Notably, the ASMR of T2DM in the 15–49 age group for males is still a concern. This indicates that early-onset T2DM has become an increasingly serious global health issue among adolescents and young adults (13). Additionally, the higher ASMR observed among females in the Eastern Mediterranean region may reflect gender disparities in healthcare access, cultural and socioeconomic constraints, and potential differences in disease awareness or exposure to risk factors (14).

There are socioeconomic disparities in the risk of diabetes mellitus-related mortality, with low- and middle-income countries bearing an increasing burden of diabetes mellitus and untreated diabetes mellitus. Patients with diabetes mellitus in low-income regions face higher mortality risks compared to those in high-income regions (15). This may be because, in most countries, especially low- and middle-income ones, access to diabetes mellitus treatment has not increased (or has not sufficiently increased relative to the rising prevalence) (16). This implies that a large number of diabetes mellitus patients in these areas are at significant risk of severe complications. We found that the highest mortality burden of diabetes mellitus is in Africa. This may be due to the fact that in most African countries, the cost of diabetes mellitus care exceeds the financial capacity of individuals, families, and governments (17). While Africa has made significant efforts over the past few decades to eliminate and control diseases and improve healthcare services, issues with diabetes mellitus diagnosis persist, with many individuals unaware they have the disease, particularly those with T2DM, who can live for extended periods without complications (18). The lack of adequate healthcare infrastructure and resources exacerbates the situation, contributing to the high mortality burden. The fastest-growing region for T2DM ASMR is expected to be South-East Asia. On the one hand, this may be due to the large number of people with impaired fasting blood sugar in South-East Asia. On the other hand, it may be related to the lower health expenditures for diabetes mellitus in South-East Asia (3). Expanding health insurance and primary healthcare, developing diabetes mellitus response plans, and adjusting healthcare services could be useful in reducing diabetes mellitus-related mortality.

The improvement of social development level is associated with the reduction of burden of T2DM. The non-communicable disease (NCD) Countdown 2030 report indicates that the global decline in diabetes mellitus-related ASMR is progressing too slowly, and in many countries, it may even be worsening to the extent that achieving the United Nations Sustainable Development Goals of reducing overall non-communicable disease ASMR by one-third by 2030 may not be feasible (19, 20). However, in many countries with a high-middle SDI, the ASMR remains significantly higher and has a lot of room for improvement. We found that age structure and population growth are the main factors contributing to the increasing absolute number of deaths of T2DM, which is mainly attributed to increasing life expectancy and the aging of the global population. The number of elderly people suffering from T2DM is still rapidly increasing, and elderly patients are highly heterogeneous, often complicated by multiple complications, and their management is complex (21). The latest report shows that by 2050, the elderly population is expected to account for more than 16% of the global population (22). Over the past three decades, for every one-unit increase in the global high fasting glucose exposure rate, the mortality rate increased by 20.9 per 100,000 individuals. It is estimated that during the period from 2022 to 2040, the impact of high fasting blood glucose in Europe will increase. Elevated levels of glycated hemoglobin beyond the target range serve as strong predictors of cardiovascular events in T2DM patients, indicating the significance of glycemic disorders in the ASMR of T2DM (23). A study conducted in the United States indicated that between 1990 and 2019, there was an increase in population of ASMR attributed to hyperglycemic crises (24). Therefore, it is recommended that individuals with T2DM aim to promptly control their blood sugar levels in the early stages of the disease, as this is crucial for reducing complications and lowering the risk of mortality (25). Increasing specific, evidence-based intervention measures in high-burden areas, such as implementing community-based screening programs and strengthening primary care management in high-burden areas, can help address the identified gaps (26).

This study also had some limitations. First, there were limitations of the GBD data itself, such as the shortage and sparseness of data for many countries and some age subgroups, although the data still encompassed more than 80% of countries and regions reporting GBD. Second, deaths in 2020 and 2021 may be affected by the COVID-19 pandemic, which will impact the projections. Third, we relied on diabetes mellitus registration system diagnosis, so our estimates were affected by differences in diagnostic practices across regions, differences in interoperability with international systems, insufficient coding rules, incomplete availability of codes, and difficulties in diagnosing comorbidities associated with these codes. Additionally, as future trends in T2DM mortality may be shaped by evolving healthcare systems, intervention strategies, and risk factor dynamics, our projections should be viewed as indicative estimates based on past patterns rather than definitive predictions. Finally, compared with the recent study by Zhou et al., which also used GBD 2021 data to project global and cross-SDI trends in T2DM-related mortality and DALYs, our analysis differs in modeling approach, decomposition framework, and regional focus, which may explain the observed similarities and discrepancies in the results (27).

## 5 Conclusion

The global mortality burden of T2DM is expected to remain concentrated in low- and middle-income countries in the future, with the death burden in both youth and elderly populations continuing to increase. Factors such as population aging, social development levels, and fasting blood glucose levels all influence the mortality burden. In conclusion, T2DM will continue to pose a threat to the global burden of non-communicable diseases, and we need to adjust healthcare services and provide healthcare resources to further reduce the mortality burden of T2DM.

## Data Availability

The datasets presented in this study can be found in online repositories. The names of the repository/repositories and accession number(s) can be found below: The datasets generated during and analyzed in the current study are available in the Global Health Data Exchange, http://ghdx.healthdata.org/gbd-2021/data-input-sources.
