# Supplementary material for: Global spatiotemporal trends and influencing factors of type 2 diabetes mellitus mortality: estimates and predictions from 1990 to 2040
Source: Front Endocrinol (Lausanne). 2025 Aug 28;16:1601089. doi: 10.3389/fendo.2025.1601089 (PMC12422939; doi:10.3389/fendo.2025.1601089)
Supplement: Supplementary file 1 [file DataSheet1.docx]

Global spatiotemporal trends and influencing factors of type 2 diabetes mellitus mortality: estimates and predictions from 1990 to 2040

Supplemental online content

Yue Zhang, Huan Deng, Jian Zu, Yujiao Deng, Jingyue Tan, Zhanpeng Yang, Yang Jiao, Xiaomeng Cui, Lei Zhang, Fanpu Ji, Yuan Wang

1. Supplementary Methods
   1. Bayesian Age-Period-Cohort model
   2. Average Annual Percent Change
   3. Decomposition analysis
   4. Frontier analysis
   5. Bayesian spatio-temporal model
   6. Bayesian spatio-temporal model with influencing factors
2. Supplementary Figures
   1. Supplementary Figure S1. The spatial pattern of type 2 diabetes mellitus age-standardized mortality rates (ASMR) across 204 countries and territories in 2021 and 2040.
3. Supplementary Tables
   1. Supplementary Table S1. The number of deaths due to type 2 diabetes mellitus across the world and six regions in 1990–2040.
   2. Supplementary Table S2. Age-standardized mortality rates and average annual percentage change in type 2 diabetes mellitus by sex and age, global and six regions in 1990–2040.
   3. Supplementary Table S3. Contributions of age structure, population growth, and epidemiological changes to the increase in T2DM deaths globally and in six regions in 1990–2021 and 2022–2040.
   4. Supplementary Table S4. The RMSE values of both Auto-ARIMA and Prophet for SDI/HFPG prediction with the results of model selection in 204 countries and territories.
   5. Supplementary Table S5. High Fasting Plasma Glucose (HFPG) exposure rates, socio-demographic index (SDI) in 1990–2040 in 204 countries and territories.
   6. Supplementary Table S6. Age-standardized mortality rates and average annual percentage changes for 1990–2040 and differences between 1990–2021 vs. 2022–2040 periods of type 2 diabetes mellitus in 204 countries and territories.
   7. Supplementary Table S7. The frontier analysis based on socio-demographic index and age-standardized mortality rate for T2DM in 2021 and 2040 in 204 countries and territories to obtain the frontier line (optimal age-standardized mortality rate corresponding to each socio-demographic index).
4. References

1. Supplementary Methods
   1. Bayesian Age-Period-Cohort model

We employed the Bayesian Age-Period-Cohort (BAPC) model [1, 2] to predict the Age-Standardized Mortality Rate (ASMR) for the global, six regions, and 204 countries (territories) from 2022 to 2040. The BAPC model was an improvement based on the Age-Period-Cohort (APC) model, which was primarily a log-linear Poisson model. For data with size $I\times J$, the rows represented $I$ age groups $\left( i=1,2,\cdots,I \right)$, the columns represented $J$ observation periods $\left( j=1,2,\cdots,J \right)$, and the diagonals represented $K$ birth cohorts $\left( k=1,2,\cdots,K \right)$. Cohort effect $K=M\left( I-i \right)+j,M=5$ (M was the width of the age bands). The observed mortality counts $y_{ij}$ in age group $i$ in period $j$ can be assumed to be Poisson distributed with mean $n_{ij}\lambda_{ij}$, where $n_{ij}$ denoted the corresponding persontime of exposure. The predictor was commonly specified as a linear combination of an intercept $\mu$, age effect $\alpha_{i}$, period effects $\pi_{j}$ and cohort effect $\gamma_{k}$:

$$\log\left( \lambda_{ij} \right)=\mu+\alpha_{i}+\pi_{j}+\gamma_{k}.$$

In our BAPC model, the effects of age, period, and cohort was a linear time trend of second-order random walk (RW2), which constrained the second differences stochastically to zero and penalizes deviations from a linear trend:

$$\alpha_{i}\sim N\left( 2\alpha_{i-1}-\alpha_{i-2},\kappa^{-1} \right), i=2,\ldots,I,$$

where $\kappa^{-1}$ denoted the variance parameter. Additionally, to adjust for potential overdispersion (we used the data from annual to five-year intervals), additional independent mean-zero Gaussian random effects $\varepsilon_{ij}$ can be added to the linear predictor $\log\left( \lambda_{ij} \right)$:

$$\log\left( \lambda_{ij} \right)=\mu+\alpha_{i}+\pi_{j}+\gamma_{k}+\varepsilon_{ij},$$

$$\varepsilon_{ij}\sim N\left( 0,\kappa^{-1} \right).$$

The methodology used integrated nested Laplace approximations (INLA) to approximate the posterior marginal distributions directly for full Bayesian inference, which was implemented in the user-friendly R-package BAPC from R-forge (http://r-forge.r-project.org/). According to age groups (15–49 years old, 50–74 years old, and ≥75 years old), the number of deaths and demographic data of each group from 1990 to 2021, along with the predicted demographic data from 2022 to 2040, were input into the BAPC model. This process aimed to obtain the number of deaths for each age group from 1990 to 2040 and subsequently calculated the age group mortality rate. Finally, the predicted standardized mortality rate for the period 2022 to 2040 was calculated.

- 1. Average Annual Percent Change

Calculation of Average Annual Percent Change (AAPC) for regions and 204 countries (territories) using Joinpoint software over the periods 1990–2021 and 2022–2040. We divided the time nodes of different trends and calculated the annual percentage change (APC) in mortality for each time period. The Joinpoint regression model adopted the log-linear regression model $\ln y=xb$. Assuming that the death rate for a given year changes based on a constant percentage of the previous year's death rate, the model used was:

$$\log\left( R_{y} \right)=b_{0}+b_{1}y,$$

where $\log\left( R_{y} \right)$ was the natural log of the rate in year $y$. The formula for calculating APC from $y$ to $y+1$ was:

$$APC=\frac{R_{y+1}-R_{y}}{R_{y}}\times100=\frac{e^{b_{0}+b_{1}\left( y+1 \right)}-e^{b_{0}+b_{1}y}}{e^{b_{0}+b_{1}y}}\times100=\left( e^{b_{1}}-1 \right)\times100.$$

Average Annual Percent Change (AAPC) was a summary measure of the trend over a interval that reflects the overall trend in mortality change over a multi-year period. It was computed as a weighted average of the APCs from the joinpoint model, with the weights equal to the length of the APC interval:

$$AAPC=\left\{ exp\left( \frac{\sum w_{i}b_{i}}{\sum w_{i}} \right)-1 \right\}\times100,$$

where $i$ indexed the segments in the desired range of years, $b_{i}$ was the slope coefficient for the $i$^th^ segment, and $w_{i}$ was the length of each segment in the range of years. In this study, the quantitative parameter of the joinpoint model was 5.

- 1. Decomposition analysis

Decomposition analysis was an analytical method used to determine how much the difference between the influencing factors of two data contributes to their overall difference. We used decomposition analyses for world and six regions of deaths to investigate the contribution of different factors to the amount of change over 1990–2021 and 2022–2040. We first used the decomposition methodology of Das Gupta [3-5] to decompose Deaths by population age structure, population growth, and epidemiologic changes (Death rate). The number of Deaths at each location was obtained from the following formula:

$${Deaths}_{ay,py,ey}=\sum_{i=1}^{20} a_{i,y}*p_{y}*e_{i,y}$$

where ${Deaths}_{ay,py,ey}$represented Deaths based on the factors of age structure, population, and Deaths rate for specific year $y$; $a_{i,y}$ represented the proportion of population for the age category $i$ of the 20 age categories in given year $y$; $p_{y}$ represented the total population in given year $y$; and $e_{i,y}$ represented Deaths rate given age category $i$ in year $y$. The contribution of each factor to the change in Deaths from 1990 to 2021 was defined by the effect of one factor changing while the other factors were held constant. For example, the effect of age structure was calculated as follows:

$$[({Deaths}_{a2021,p1990,e1990}+{Deaths}_{a2021,p2021,e2021})/3+({Deaths}_{a2021,p1990,e2021}+{Deaths}_{a2021,p2021,e1990})/6]-[({Deaths}_{a1990,p2021,e2021}+{Deaths}_{a1990,p1990,e1990})/3+({Deaths}_{a1990,p2021,e1990}+{Deaths}_{a1990,p1990,e2021})/6]$$

Then the contribution proportion of influence factors can be calculated the effect of age structure$/({Deaths}_{a2021,p2021,e2021}-{Deaths}_{a1990,p1990,e1990})*100.$Similar equations can be used to find the contribution of the total population and the Deaths rate. The same applied to 2022–2040.

- 1. Frontier analysis

We conducted a frontier analysis of the projected ASMR for type 2 diabetes mellitus in 2040 to gain insight into the minimum mortality levels that could be achieved in 204 countries and territories at different levels of development, as well as the improvements that could be achieved under each country's development status. The method measured the development status of countries in terms of the SDI and used it to determine minimum possible ASMR for each country. We used DEA [6-8] to do the frontier analysis and FDH to construct the frontier surface. The absolute distance from each country's ASMR to the frontier surface was called the effective difference, and countries with a ASMR below and equal the frontier value were defined as zero distance, we used the effective distance to measure the country's potential for improvement in type 2 diabetes mellitus deaths.

- 1. Bayesian spatio-temporal model

Further we constructed a Bayesian spatio-temporal model [9-13] to simultaneously analyze the spatial distribution and temporal trend of ASMR in 204 countries globally from 1990–2040. Three Bayesian spatio-temporal models were used, where $y_{it}$denoted the ASMR in year$t$(2022,2023...2040) of the *i*^th^ ($i$=1,2,...204) country, let $y_{it}$ followed a normal distribution and $y_{it} \sim Normal (\mu_{it} ,\sigma^{2})$, then $\mu_{it}$ can be expressed as the formula:

$$\mu_{it}=\alpha+S_{i}+\theta_{t}+\delta_{it}$$

$\alpha$ was an intercept that measures the overall $y_{it}$ during 2022–2040，the spatial term $S_{i}$ denoted the spatial random effect capturing the spatial dependency of $y_{it}$. We noted that $S_{i}$ described the difference between $y_{it}$in the $i$^th^ country or region relative to the global average level $\alpha$, if $S_{i}>0$, indicating the ASMR in country $i$ was higher than overall ASMR across the whole study periods. The term $\theta_{t}$ denoted a dynamic temporal trend that captured the overall temporal trend common to all countries. The $\delta_{it}$ was a space-time interaction random effect, representing a vector that varies through space and time, it allowed each country to have its own temporal trend. A growing trend of the parameter $\delta_{it}$ indicated that the upward trend of the $i$^th^ country was stronger than the overall upward trend or that the downward trend was weaker than the overall downward trend, and a decreasing trend was just the opposite. We similarly used a Bayesian spatio-temporal model to describe the situation over 1990–2021 and 1990–2040. The models were implemented by the INLA package [14] in R software

- 1. Bayesian spatio-temporal model with influencing factors

A Bayesian spatio-temporal model was again used to determine the impact of SDI and High Fasting Glucose on type 2 diabetes mellitus mortality across regions. Before that, we first used Auto-ARIMA and Prophet models to predict the High Fasting Glucose exposure rate and SDI in 204 countries from 2022 to 2040, using the data from 1990 to 2019 as the training set, and the data from 2020 to 2021 as the verification set, compared the RMSE of the two models on the validation set, and finally selected the most appropriate model for prediction. The Bayesian space-time model adding influencing factors was:

$$y_{it} \sim Normal (\mu_{it} ,\sigma^{2})$$

$$\mu_{it}=\alpha+S_{i}+\theta_{t}+\delta_{it}+\sum_{j=1}^{n} \beta_{1j}X_{1jit}+\sum_{j=1}^{n} \beta_{2j}X_{2jit}+\sum_{j=1}^{n} \beta_{3j}X_{1jit}*X_{2jit}$$

Where $S_{i}$, $\theta_{t}$, $\delta_{it}$ were consistent with the above, $\beta_{ij}, i=1,2$ represented the influence of the $i$^th^ factor on the $j$^th^ country, and $\beta_{3j}$represented the interactive influence of two influencing factors on the $j$^th^ country. The measurements of the two variables were adjusted to a common scale by normalizing them using the Z-Score normalization method.

1. Supplementary Figure
   1. Supplementary Figure S1. The spatial pattern of type 2 diabetes mellitus age-standardized mortality rates (ASMR) across 204 countries and territories in 2021 and 2040. (a) ASMR across 204 countries and territories in 2021, (b) ASMR across 204 countries and territories in 2040.


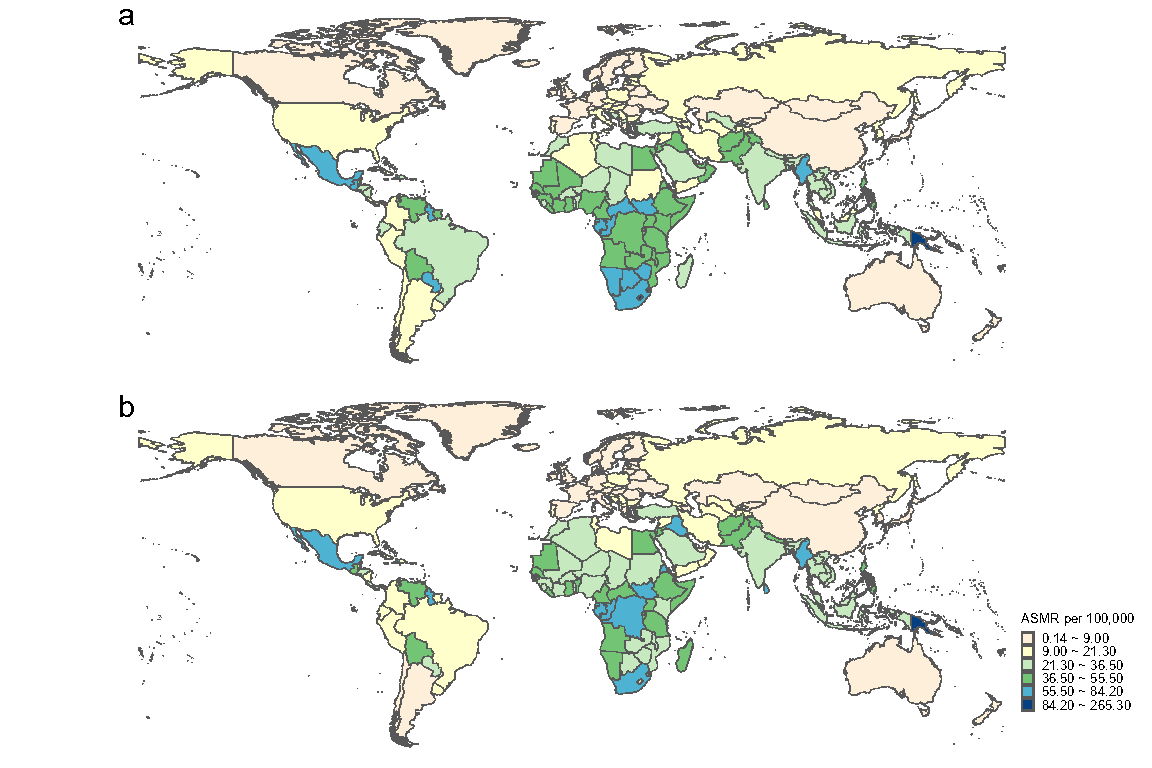


1. Supplementary Tables
   1. Supplementary Table S1. The number of deaths due to type 2 diabetes mellitus across the world and six regions in 1990–2040.

| **Region** | **1990 Deaths** | **2021 Deaths** | **2040 Deaths** |
| --- | --- | --- | --- |
| Global | 632,322 (596,870–662,082) | 1,608,123 (1,493,438–1,708,294) | 2,756,631 |
| African Region | 74,937 (68,636–82,778) | 188,903 (171,869–207,349) | 459,189 |
| Region of the Americas | 135,313 (128,236–139,071) | 302,120 (278,249–320,733) | 551,148 |
| South-East Asia Region | 141,646 (127,237–156,707) | 491,202 (445,841–540,161) | 1,035,666 |
| European Region | 124,743 (116,062–129,662) | 199,457 (179,196–211,579) | 244,272 |
| Eastern Mediterranean Region | 36,285 (32,991–40,872) | 132,896 (116,991–147,544) | 296,002 |
| Western Pacific Region | 111,500 (101,200–121,235) | 278,106 (244,524–314,039) | 498,776 |

- 1. Supplementary Table S2. Age-standardized mortality rates and average annual percentage change in type 2 diabetes mellitus by sex and age, global and six regions in 1990–2040.

| **Region** | **Sex** | **Age** | **1990 ASMR** | **2021 ASMR** | **2040 ASMR** | **1990–2021  AAPC** | **2022–2040  AAPC** |
| --- | --- | --- | --- | --- | --- | --- | --- |
| Global | Both | All ages | 17.33 (16.20–18.18) | 19.02 (17.57–20.20) | 18.63 (8.82–28.43) | 0.31 (0.21–0.42) | -0.16 (-0.17–-0.15) |
|  |  | 15–49 | 2.05 (1.91–2.19) | 2.32 (2.11–2.54) | 2.35 (1.32–3.93) | 0.40  (0.28–0.52) | 0.19  (0.14–0.25) |
|  |  | 50–74 | 47.65 (45.11–50.21) | 51.02 (47.69–54.34) | 51.58 (29.00–84.55) | 0.23  (0.09–0.38) | 0.14  (0.09–0.18) |
|  |  | ≥75 | 211.85 (189.32–225.53) | 237.96 (209.03–256.09) | 237.48 (133.44–389.07) | 0.41  (0.21–0.62) | -0.10  (-0.13–-0.07) |
|  | Female | All ages | 16.94 (15.66–17.9) | 18.03 (16.54–19.29) | 17.29 (7.33–27.25) | 0.21 (0.09–0.33) | -0.26 (-0.27–-0.24) |
|  |  | 15–49 | 1.93 (1.76–2.11) | 2.06 (1.85–2.27) | 2.01 (1.07–3.53) | 0.23  (0.04–0.43) | 0.05  (-0.01–0.1) |
|  |  | 50–74 | 46.89 (43.81–50.08) | 48.25 (44.69–51.91) | 48.25 (25.68–82.48) | 0.09  (-0.11–0.3) | 0.10  (0.06–0.14) |
|  |  | ≥75 | 206.4 (180.63–222.01) | 227.94 (196.58–248.16) | 224.26 (119.29–383.11) | 0.35  (0.17–0.53) | -0.17  (-0.17–-0.16) |
|  | Male | All ages | 17.89 (16.69–19.1) | 20.27 (18.67–21.86) | 20.30 (10.33–30.27) | 0.45 (0.32–0.58) | -0.05 (-0.07–-0.04) |
|  |  | 15–49 | 2.17 (1.98–2.37) | 2.57 (2.29–2.89) | 2.78 (1.62–4.5) | 0.57  (0.46–0.67) | 0.48  (0.44–0.52) |
|  |  | 50–74 | 48.46 (45.08–52.15) | 53.97 (49.4–58.46) | 55.13 (32.13–87.56) | 0.36  (0.17–0.55) | 0.19  (0.15–0.23) |
|  |  | ≥75 | 221.73 (201.17–238.73) | 254.28 (226.7–275.65) | 258.42 (150.54–410.26) | 0.45  (0.3–0.6) | -0.02  (-0.03–-0.01) |
| African Region | Both | All ages | 38.65 (35.25–42.59) | 44.19 (40.47–48.14) | 41.68 (25.65–57.72) | 0.43 (0.32–0.53) | -0.376 (-0.379–-0.372) |
|  |  | 15–49 | 4.61 (3.98–5.29) | 4.34 (3.7–5.06) | 3.97 (2.68–5.67) | -0.2  (-0.33–-0.07) | -0.45  (-0.45–-0.44) |
|  |  | 50–74 | 108.41 (97.77–121.23) | 113.59 (102.21–126.54) | 105.00 (71.4–149.34) | 0.16  (0.03–0.28) | -0.42  (-0.42–-0.41) |
|  |  | ≥75 | 460.9 (403.01–516.41) | 592.54 (527.38–646.88) | 568.50 (386.57–808.57) | 0.83  (0.69–0.97) | -0.27  (-0.28–-0.27) |
|  | Female | All ages | 34.53 (30.75–38.37) | 41.07 (37.39–45.38) | 36.81 (20.4–53.22) | 0.55 (0.37–0.73) | -0.66 (-0.67–-0.65) |
|  |  | 15–49 | 4.06 (3.31–4.76) | 3.47 (2.85–4.03) | 3.07 (1.86–4.84) | -0.53  (-0.72–-0.33) | -0.45  (-0.47–-0.44) |
|  |  | 50–74 | 94.97 (83.33–108.5) | 102.58 (90.99–115.71) | 86.68 (52.87–135.46) | 0.26  (0.04–0.47) | -0.863  (-0.866–-0.860) |
|  |  | ≥75 | 421.88 (358.4–479.53) | 573.43 (499.77–635.02) | 549.78 (335.34–859.18) | 1.00  (0.8–1.2) | -0.330  (-0.335–-0.326) |
|  | Male | All ages | 43.27 (38.46–49.95) | 47.82 (42.7–52.52) | 46.88 (29.08–64.67) | 0.34 (0.26–0.41) | -0.151 (-0.152–-0.150) |
|  |  | 15–49 | 5.14 (4.36–6.2) | 5.25 (4.37–6.3) | 4.99 (3.41–7.04) | 0.03  (-0.07–0.14) | -0.37  (-0.38–-0.37) |
|  |  | 50–74 | 121.88 (105.45–142.28) | 125.81 (110.36–142.34) | 126.02 (86.49–177.13) | 0.12  (-0.01–0.25) | -0.02  (-0.03–-0.02) |
|  |  | ≥75 | 513.99 (435.55–598.45) | 619.15 (539.41–692.53) | 599.3 (411.29–842.34) | 0.62  (0.58–0.66) | -0.178  (-0.181–-0.175) |
| Region of the Americas | Both | All ages | 22.41 (21.18–23.06) | 22.27 (20.53–23.64) | 23.04 (0.68–45.41) | 0.03 (-0.20–026) | 0.01 (0.007–0.015) |
|  |  | 15–49 | 3.13 (3.06–3.2) | 3.22 (3.01–3.45) | 2.88 (0.95–7) | 0.22  (-0.05–0.48) | -0.14  (-0.2–-0.08) |
|  |  | 50–74 | 65.05 (62.92–66.68) | 62.03 (58.2–65.87) | 74 (23.86–174.32) | -0.11  (-0.38–0.16) | 0.91  (0.89–0.93) |
|  |  | ≥75 | 249.7 (221.86–264.41) | 260.13 (225.9–280.76) | 305.84 (98.54–719.92) | 0.15  (0.03–0.28) | 0.52  (0.47–0.56) |
|  | Female | All ages | 22.56 (21.1–23.36) | 20.25 (18.36–21.69) | 20.62 (1.07–40.18) | -0.32 (-0.54–-0.09) | -0.021 (-0.028–-0.015) |
|  |  | 15–49 | 2.94 (2.86–3.03) | 2.73 (2.49–2.98) | 2.37 (0.81–5.77) | -0.11  (-0.36–0.14) | -0.35  (-0.41–-0.28) |
|  |  | 50–74 | 65.9 (63.25–67.99) | 54.23 (50.24–58.15) | 65.34 (21.74–152.47) | -0.62  (-0.9–-0.34) | 1.06  (1.05–1.08) |
|  |  | ≥75 | 252.12 (219.71–269.28) | 250.08 (211.98–273.8) | 274.82 (91.34–640.62) | 0.02  (-0.23–0.26) | 0.26  (0.23–0.29) |
|  | Male | All ages | 22.17 (21.26–22.71) | 24.6 (22.72–26.28) | 24.64 (-0.43–49.7) | 0.41 (0.16–0.67) | -0.106 (-0.108–-0.105) |
|  |  | 15–49 | 3.32 (3.22–3.42) | 3.74 (3.47–4.06) | 3.72 (1.14–9.19) | 0.54  (0.28–0.79) | 0.43  (0.4–0.47) |
|  |  | 50–74 | 64.05 (62.14–65.77) | 70.73 (65.56–76.31) | 80.28 (24.37–195.01) | 0.4  (0.12–0.67) | 0.74  (0.71–0.78) |
|  |  | ≥75 | 245.64 (224.74–257.54) | 273.53 (242.16–293.99) | 319.09 (96.83–774.85) | 0.39  (0.27–0.51) | 0.54  (0.46–0.61) |
| South-East Asia Region | Both | All ages | 23.57 (20.97–26.12) | 30.54 (27.74–33.64) | 32.26 (9.78–54.74) | 0.88 (0.43–1.34) | 0.222 (0.216–0.228) |
|  |  | 15–49 | 2.61 (2.28–2.93) | 2.73 (2.38–3.13) | 2.32 (1.18–4.13) | 0.17  (0.08–0.27) | -0.88  (-0.89–-0.86) |
|  |  | 50–74 | 66.93 (59.27–74.66) | 77.82 (69.57–86) | 86.6 (44.18–153.3) | 0.49  (0.14–0.84) | 0.57  (0.57–0.58) |
|  |  | ≥75 | 279.82 (237.54–320.71) | 416.73 (364.73–464.67) | 494.04 (252.03–874.56) | 1.29  (0.69–1.89) | 0.89  (0.86–0.92) |
|  | Female | All ages | 22.88 (19.76–26.19) | 29.79 (26.64–33.23) | 29.7 (5.75–53.65) | 0.87 (0.38–1.37) | -0.11 (-0.12–-0.10) |
|  |  | 15–49 | 2.67 (2.25–3.15) | 2.62 (2.22–3.06) | 2.05 (0.95–3.84) | -0.02  (-0.25–0.2) | -1.14  (-1.15–-1.13) |
|  |  | 50–74 | 65.81 (56.33–76.12) | 76.36 (67.52–86.51) | 82.58 (38.65–153.48) | 0.48  (0.09–0.86) | 0.38  (0.38–0.38) |
|  |  | ≥75 | 265.37 (217.35–316.13) | 404.65 (344.73–462.24) | 489.8 (229.27–910.35) | 1.46  (0.75–2.18) | 0.96  (0.93–0.98) |
|  | Male | All ages | 24.35 (20.58–27.84) | 31.52 (27.63–36.38) | 33.68 (11.28–56.08) | 0.80 (0.60–1.02) | 0.331 (0.328–0.335) |
|  |  | 15–49 | 2.54 (2.16–2.96) | 2.84 (2.33–3.43) | 2.77 (1.44–4.92) | 0.40  (0.2–0.6) | -0.28  (-0.3–-0.26) |
|  |  | 50–74 | 68.08 (57.29–79.09) | 79.39 (67.59–92.14) | 89.34 (46.63–158.06) | 0.61  (0.21–1.01) | 0.71  (0.71–0.72) |
|  |  | ≥75 | 296.45 (237.39–357.01) | 434.6 (372.53–501.35) | 494.92 (258.31–875.61) | 1.27  (0.58–1.96) | 0.74  (0.72–0.76) |
| European Region | Both | All ages | 11.76 (10.87–12.24) | 11.02 (10.01–11.67) | 8.91 (-1.42–19.25) | -0.21 (-0.40–-0.01) | -1.13 (-1.14–-1.11) |
|  |  | 15–49 | 0.79 (0.74–0.84) | 0.74 (0.69–0.8) | 0.82 (0.19–2.37) | -0.22  (-0.5–0.06) | 0.40  (0.34–0.46) |
|  |  | 50–74 | 27.67 (26.45–28.88) | 25.48 (23.87–27.19) | 23.39 (5.38–67.78) | -0.26  (-0.43–-0.09) | -0.25  (-0.29–-0.2) |
|  |  | ≥75 | 175.89 (154.97–188.27) | 167.3 (141.91–181.87) | 160.45 (36.88–464.86) | -0.13  (-0.38–0.11) | -0.12  (-0.15–-0.08) |
|  | Female | All ages | 11.54 (10.52–12.07) | 10.18 (9–10.89) | 8.62 (-2.63–19.87) | -0.40 (-0.76–-0.04) | -0.83 (-0.85–-0.80) |
|  |  | 15–49 | 0.64 (0.58–0.71) | 0.54 (0.49–0.61) | 0.68 (0.12–2.21) | -0.46  (-0.9–-0.02) | 0.91  (0.83–0.99) |
|  |  | 50–74 | 26.66 (25.19–28.09) | 22.23 (20.42–24.04) | 21.43 (3.97–69.26) | -0.65  (-1–-0.29) | 0.25  (0.12–0.37) |
|  |  | ≥75 | 177.04 (152.55–192.28) | 163.2 (134.78–180.76) | 154.7 (28.67–500.03) | -0.25  (-0.64–0.15) | -0.01  (-0.06–0.05) |
|  | Male | All ages | 11.93 (11.33–12.4) | 12.04 (11.17–12.67) | 9.43 (0.08–18.78) | 0.03 (-0.21–0.28) | -1.368 (-1.374–-1.357) |
|  |  | 15–49 | 0.94 (0.87–1.02) | 0.94 (0.87–1.02) | 0.97 (0.24–2.66) | -0.04  (-0.36–0.28) | 0.12  (0.05–0.18) |
|  |  | 50–74 | 28.9 (27.44–30.83) | 29.23 (27.08–31.59) | 25.97 (6.52–71.11) | 0.04  (-0.25–0.33) | -0.56  (-0.6–-0.52) |
|  |  | ≥75 | 172.12 (156.82–183.07) | 173.71 (152.22–188.1) | 173.53 (43.58–475.2) | 0.04  (-0.24–0.32) | -0.13  (-0.16–-0.11) |
| Eastern Mediterranean Region | Both | All ages | 23.39 (21.05–26.5) | 34.71 (30.7–38.36) | 34.87 (15–54.73) | 1.30 (1.19–1.41) | -0.07 (-0.074–-0.067) |
|  |  | 15–49 | 2.32 (1.99–2.69) | 2.91 (2.44–3.44) | 3.27 (1.75–5.6) | 0.75  (0.68–0.82) | 0.56  (0.54–0.57) |
|  |  | 50–74 | 62.93 (55.89–71.39) | 91.75 (78.74–105.1) | 88.59 (47.49–151.07) | 1.24  (1.09–1.38) | -0.14  (-0.21–-0.07) |
|  |  | ≥75 | 298.83 (251.29–346.09) | 459.05 (392.8–521.15) | 480.4 (257.51–819.19) | 1.41  (1.26–1.57) | 0.22  (0.22–0.22) |
|  | Female | All ages | 25.81 (23.35–28.27) | 38.8 (33.87–44.09) | 37.21 (15.9–58.51) | 1.34 (1.20–1.48) | -0.385 (-0.388–-0.382) |
|  |  | 15–49 | 2.51 (2.09–2.99) | 3.17 (2.49–3.91) | 3.52 (1.89–6.01) | 0.76  (0.64–0.89) | 0.53  (0.51–0.56) |
|  |  | 50–74 | 70.1 (60.95–79.66) | 100.34 (83.54–119.49) | 92.3 (49.82–156.48) | 1.18  (1.01–1.34) | -0.36  (-0.38–-0.35) |
|  |  | ≥75 | 327.06 (270.87–386.4) | 525.5 (436.29–612.34) | 507.79 (274.04–860.79) | 1.55  (1.38–1.73) | -0.25  (-0.27–-0.23) |
|  | Male | All ages | 21.26 (18.55–26.89) | 30.88 (26.53–35.26) | 32.58 (14.86–50.30) | 1.22 (1.07–1.38) | 0.227 (0.222–0.230) |
|  |  | 15–49 | 2.13 (1.76–2.7) | 2.68 (2.16–3.25) | 2.99 (1.59–5.16) | 0.77  (0.69–0.84) | 0.48  (0.47–0.5) |
|  |  | 50–74 | 56.69 (47.67–71.58) | 83.71 (69.5–99.41) | 85.8 (45.71–147.36) | 1.28  (1.17–1.39) | 0.14  (0.04–0.24) |
|  |  | ≥75 | 273.94 (216.39–352.8) | 396.71 (328.46–467.55) | 455.5 (242.69–782.27) | 1.22  (1.05–1.39) | 0.75  (0.74–0.76) |
| Western Pacific Region | Both | All ages | 11.1 (10.02–12.03) | 9.92 (8.72–11.2) | 9.79 (1.87–17.71) | -0.36 (-0.50–-0.23) | -0.12 (-0.13–-0.11) |
|  |  | 15–49 | 1.23 (1.09–1.38) | 1.21 (1.05–1.38) | 1.57 (0.65–3.24) | -0.07  (-0.25–0.11) | 1.55  (1.45–1.64) |
|  |  | 50–74 | 29.95 (26.86–33.18) | 25.45 (22.21–29.06) | 28.4 (11.87–58.15) | -0.52  (-0.62–-0.41) | 0.57  (0.55–0.59) |
|  |  | ≥75 | 139.68 (122.3–154.18) | 130.02 (109.26–148.26) | 138.04 (57.71–282.65) | -0.24  (-0.45–-0.02) | 0.16  (0.11–0.21) |
|  | Female | All ages | 11.42 (10.01–12.77) | 9.39 (8.05–10.82) | 8.92 (1.27–16.56) | -0.65 (-0.78–-0.52) | -0.287 (-0.291–-0.283) |
|  |  | 15–49 | 1.06 (0.89–1.27) | 0.93 (0.79–1.08) | 1.27 (0.52–2.64) | -0.43  (-0.63–-0.23) | 1.81  (1.75–1.88) |
|  |  | 50–74 | 32.35 (27.8–37.67) | 24.96 (21.07–29.62) | 26.47 (11.05–53.96) | -0.82  (-0.9–-0.75) | 0.40  (0.38–0.42) |
|  |  | ≥75 | 138.87 (116.71–158.89) | 121.5 (96.87–143.22) | 123.31 (51.48–251.39) | -0.42  (-0.65–-0.2) | -0.07  (-0.09–-0.05) |
|  | Male | All ages | 10.82 (9.66–12.07) | 10.71 (9.13–12.54) | 10.64 (2.26–19.02) | -0.04 (-018–0.10) | -0.105 (-0.112–-0.098) |
|  |  | 15–49 | 1.39 (1.19–1.61) | 1.47 (1.24–1.75) | 1.81 (0.77–3.63) | 0.19  (0.07–0.32) | 1.22  (1.17–1.27) |
|  |  | 50–74 | 27.32 (23.82–31.51) | 25.92 (21.46–31.17) | 28.73 (12.42–57.34) | -0.16  (-0.26–-0.06) | 0.49  (0.46–0.51) |
|  |  | ≥75 | 143.16 (127.3–159.98) | 145.87 (121.74–171.01) | 158.66 (68.61–316.69) | 0.02  (-0.21–0.26) | 0.24  (0.21–0.28) |

- 1. Supplementary Table S3. Contributions of age structure, population growth, and epidemiological changes to the increase in T2DM deaths globally and in six regions in 1990–2021 and 2022–2040.

| **Region** | **1990–2021**  **Age** | **1990–2021 Population** | **1990–2021 Epidemiologic** | **2022–2040**  **Age** | **2022–2040 Population** | **2022–2040 Epidemiologic** |
| --- | --- | --- | --- | --- | --- | --- |
| Global | 48.04% | 41.96% | 10.00% | 68.72% | 30.28% | 1.01% |
| South-East Asia Region | 45.61% | 36.86% | 17.53% | 70.02% | 16.79% | 13.19% |
| Region of the Americas | 56.02% | 44.99% | -1.00% | 63.37% | 16.23% | 20.39% |
| European Region | 96.08% | 18.09% | -14.16% | 118.43% | -7.67% | -10.76% |
| Western Pacific Region | 88.22% | 25.35% | -13.57% | 91.98% | -0.65% | 8.67% |
| African Region | 2.31% | 88.04% | 9.65% | 45.82% | 64.23% | -10.05% |
| Eastern Mediterranean Region | 17.84% | 52.64% | 29.52% | 58.89% | 38.63% | 2.47% |

- 1. Supplementary Table S4. The RMSE values of both Auto-ARIMA and Prophet for SDI/HFPG prediction with the results of model selection in 204 countries and territories.

| **Country (Territory)** | **High Fasting Plasma Glucose** | | | **Socio-Demographic Index** | | |
| --- | --- | --- | --- | --- | --- | --- |
|  | **Auto-ARIMA'RMSE** | **Prophet'RMSE** | **Model selection** | **Auto-ARIMA'RMSE** | **Prophet'RMSE** | **Model selection** |
| Afghanistan | 0.1816 | 0.4281 | Auto-ARIMA | 0.0003 | 0.0009 | Auto-ARIMA |
| Albania | 0.0793 | 0.0597 | Prophet | 0.0018 | 0.0036 | Auto-ARIMA |
| Algeria | 0.0351 | 0.0191 | Prophet | 0.0003 | 0.0014 | Auto-ARIMA |
| American Samoa | 0.213 | 0.1197 | Prophet | 0.0014 | 0.0039 | Auto-ARIMA |
| Andorra | 0.05 | 0.0443 | Prophet | 0.0019 | 0.0013 | Prophet |
| Angola | 0.0177 | 0.0163 | Prophet | 0.0006 | 0.0016 | Auto-ARIMA |
| Antigua and Barb. | 0.0445 | 0.0377 | Prophet | 0.0013 | 0.0009 | Prophet |
| Argentina | 0.1892 | 0.1092 | Prophet | 0.0038 | 0.0066 | Auto-ARIMA |
| Armenia | 0.1564 | 0.2131 | Auto-ARIMA | 0.0018 | 0.0005 | Prophet |
| Australia | 0.055 | 0.0097 | Prophet | 0.0022 | 0.0017 | Prophet |
| Austria | 0.0069 | 0.0135 | Auto-ARIMA | 0.0019 | 0.0007 | Prophet |
| Azerbaijan | 0.1298 | 0.0981 | Prophet | 0.0016 | 0.002 | Auto-ARIMA |
| Bahamas | 0.0458 | 0.0458 | Auto-ARIMA | 0.0011 | 0.0011 | Auto-ARIMA |
| Bahrain | 0.1205 | 0.2057 | Auto-ARIMA | 0.0018 | 0.0033 | Auto-ARIMA |
| Bangladesh | 0.0102 | 0.3594 | Auto-ARIMA | 0.0026 | 0.0043 | Auto-ARIMA |
| Barbados | 0.0666 | 0.0242 | Prophet | 0.0015 | 0.0001 | Prophet |
| Belarus | 0.0433 | 0.0717 | Auto-ARIMA | 0.0039 | 0.0073 | Auto-ARIMA |
| Belgium | 0.024 | 0.048 | Auto-ARIMA | 0.002 | 0.0029 | Auto-ARIMA |
| Belize | 0.1412 | 0.0789 | Prophet | 0.0014 | 0.0026 | Auto-ARIMA |
| Benin | 0.0929 | 0.1239 | Auto-ARIMA | 0.0012 | 0.0007 | Prophet |
| Bermuda | 0.0688 | 0.0327 | Prophet | 0.0001 | 0.0011 | Auto-ARIMA |
| Bhutan | 0.3539 | 0.3421 | Prophet | 0.0012 | 0.0044 | Auto-ARIMA |
| Bolivia | 0.0551 | 0.0343 | Prophet | 0.0016 | 0.0034 | Auto-ARIMA |
| Bosnia and Herz. | 0.0239 | 0.0063 | Prophet | 0.0035 | 0.0006 | Prophet |
| Botswana | 0.009 | 0.014 | Auto-ARIMA | 0.0015 | 0.0027 | Auto-ARIMA |
| Brazil | 0.3344 | 0.5771 | Auto-ARIMA | 0.0011 | 0.0007 | Prophet |
| Brunei | 0.1049 | 0.0393 | Prophet | 0.0003 | 0.0013 | Auto-ARIMA |
| Bulgaria | 0.0704 | 0.085 | Auto-ARIMA | 0.0009 | 0.0009 | Auto-ARIMA |
| Burkina Faso | 0.1204 | 0.1378 | Auto-ARIMA | 0.0012 | 0.0006 | Prophet |
| Burundi | 0.0294 | 0.0225 | Prophet | 0.001 | 0.0026 | Auto-ARIMA |
| Côte d'Ivoire | 0.0746 | 0.1052 | Auto-ARIMA | 0.0008 | 0.0022 | Auto-ARIMA |
| Cabo Verde | 0.1704 | 0.2297 | Auto-ARIMA | 0.0031 | 0.0015 | Prophet |
| Cambodia | 0.1841 | 0.1238 | Prophet | 0.0021 | 0.0011 | Prophet |
| Cameroon | 0.1103 | 0.088 | Prophet | 0.0013 | 0.0005 | Prophet |
| Canada | 0.0369 | 0.0833 | Auto-ARIMA | 0.0024 | 0.0023 | Prophet |
| Central African Rep. | 0.0866 | 0.2784 | Auto-ARIMA | 0.0007 | 0.0058 | Auto-ARIMA |
| Chad | 0.0047 | 0.055 | Auto-ARIMA | 0.0007 | 0.0016 | Auto-ARIMA |
| Chile | 0.3079 | 0.2883 | Prophet | 0.0045 | 0.0081 | Auto-ARIMA |
| China | 0.1872 | 0.2489 | Auto-ARIMA | 0.0006 | 0.0092 | Auto-ARIMA |
| Colombia | 0.0824 | 0.236 | Auto-ARIMA | 0.0012 | 0.0035 | Auto-ARIMA |
| Comoros | 0.0397 | 0.028 | Prophet | 0.0017 | 0.0025 | Auto-ARIMA |
| Cook Is. | 0.1699 | 0.1504 | Prophet | 0.0025 | 0.0025 | Prophet |
| Costa Rica | 0.3062 | 0.1935 | Prophet | 0.0035 | 0.0021 | Prophet |
| Croatia | 0.0712 | 0.0636 | Prophet | 0.0017 | 0.0008 | Prophet |
| Cuba | 0.1299 | 0.2323 | Auto-ARIMA | 0.0024 | 0.0025 | Auto-ARIMA |
| Cyprus | 0.1226 | 0.193 | Auto-ARIMA | 0.0007 | 0.0015 | Auto-ARIMA |
| Czechia | 0.1961 | 0.2297 | Auto-ARIMA | 0.0006 | 0.0026 | Auto-ARIMA |
| Dem. Rep. Korea | 0.06 | 0.095 | Auto-ARIMA | 0.0001 | 0.0039 | Auto-ARIMA |
| Democratic Republic of the Congo | 1.4351 | 0.0763 | Prophet | 0.003 | 0.0049 | Auto-ARIMA |
| Denmark | 0.0868 | 0.1399 | Auto-ARIMA | 0.0028 | 0.0015 | Prophet |
| Djibouti | 0.0346 | 0.0164 | Prophet | 0.0018 | 0.0008 | Prophet |
| Dominica | 0.1814 | 0.0683 | Prophet | 0.0011 | 0.0074 | Auto-ARIMA |
| Dominican Rep. | 0.008 | 0.0709 | Auto-ARIMA | 0.0022 | 0.0012 | Prophet |
| Ecuador | 0.1304 | 0.0109 | Prophet | 0.002 | 0.0024 | Auto-ARIMA |
| Egypt | 0.0877 | 0.2677 | Auto-ARIMA | 0.0004 | 0.0045 | Auto-ARIMA |
| El Salvador | 0.3693 | 0.2664 | Prophet | 0.0012 | 0.0022 | Auto-ARIMA |
| Eq. Guinea | 1.1484 | 1.1983 | Auto-ARIMA | 0.0028 | 0.0057 | Auto-ARIMA |
| Eritrea | 0.0244 | 0.028 | Auto-ARIMA | 0.0021 | 0.0013 | Prophet |
| Estonia | 0.087 | 0.0691 | Prophet | 0.0013 | 0.0015 | Auto-ARIMA |
| eSwatini | 0.1416 | 0.119 | Prophet | 0.0009 | 0.0027 | Auto-ARIMA |
| Ethiopia | 0.043 | 0.0593 | Auto-ARIMA | 0.0031 | 0.0061 | Auto-ARIMA |
| Fiji | 0.0293 | 0.2045 | Auto-ARIMA | 0.0009 | 0.0028 | Auto-ARIMA |
| Finland | 0.0933 | 0.0571 | Prophet | 0.0017 | 0.0026 | Auto-ARIMA |
| France | 0.0289 | 0.0725 | Auto-ARIMA | 0.0016 | 0.002 | Auto-ARIMA |
| Gabon | 0.3839 | 0.131 | Prophet | 0.0013 | 0.0004 | Prophet |
| Gambia | 0.0596 | 0.0642 | Auto-ARIMA | 0.0009 | 0.0006 | Prophet |
| Georgia | 0.2249 | 0.266 | Auto-ARIMA | 0.0023 | 0.0025 | Auto-ARIMA |
| Germany | 0.1104 | 0.1041 | Prophet | 0.002 | 0.0007 | Prophet |
| Ghana | 0.1774 | 0.1712 | Prophet | 0.002 | 0.0008 | Prophet |
| Greece | 0.0153 | 0.1883 | Auto-ARIMA | 0.0007 | 0.0028 | Auto-ARIMA |
| Greenland | 0.2317 | 0.2331 | Auto-ARIMA | 0.0004 | 0.0015 | Auto-ARIMA |
| Grenada | 0.0398 | 0.0177 | Prophet | 0.002 | 0.0018 | Prophet |
| Guam | 0.16 | 0.2132 | Auto-ARIMA | 0.0012 | 0.0013 | Auto-ARIMA |
| Guatemala | 0.1415 | 0.0756 | Prophet | 0.0024 | 0.0009 | Prophet |
| Guinea | 0.2261 | 0.3158 | Auto-ARIMA | 0.0019 | 0.0008 | Prophet |
| Guinea-Bissau | 0.0241 | 0.022 | Prophet | 0.0015 | 0.0004 | Prophet |
| Guyana | 0.2797 | 0.1908 | Prophet | 0.0038 | 0.0042 | Auto-ARIMA |
| Haiti | 0.0139 | 0.0665 | Auto-ARIMA | 0.001 | 0.003 | Auto-ARIMA |
| Honduras | 0.1601 | 0.1565 | Prophet | 0.0017 | 0.0017 | Auto-ARIMA |
| Hungary | 0.1781 | 0.1359 | Prophet | 0.0002 | 0.0018 | Auto-ARIMA |
| Iceland | 0.0272 | 0.0175 | Prophet | 0.0023 | 0.0037 | Auto-ARIMA |
| India | 0.1645 | 0.0929 | Prophet | 0.0015 | 0.0069 | Auto-ARIMA |
| Indonesia | 0.3597 | 1.0382 | Auto-ARIMA | 0.0012 | 0.0026 | Auto-ARIMA |
| Iran | 0.1225 | 0.0899 | Prophet | 0.0002 | 0.0007 | Auto-ARIMA |
| Iraq | 0.3035 | 0.6092 | Auto-ARIMA | 0.0014 | 0.0039 | Auto-ARIMA |
| Ireland | 0.0214 | 0.1479 | Auto-ARIMA | 0.0043 | 0.0043 | Prophet |
| Israel | 0.1155 | 0.0164 | Prophet | 0.0019 | 0.0006 | Prophet |
| Italy | 0.1756 | 0.3077 | Auto-ARIMA | 0.0017 | 0.0005 | Prophet |
| Jamaica | 0.0216 | 0.0153 | Prophet | 0.0014 | 0.002 | Auto-ARIMA |
| Japan | 0.0998 | 0.3105 | Auto-ARIMA | 0.0014 | 0.0007 | Prophet |
| Jordan | 1.1721 | 0.1657 | Prophet | 0.0008 | 0.0008 | Prophet |
| Kazakhstan | 0.0243 | 0.0734 | Auto-ARIMA | 0.0024 | 0.0043 | Auto-ARIMA |
| Kenya | 0.0895 | 0.0316 | Prophet | 0.0017 | 0.0008 | Prophet |
| Kiribati | 0.0367 | 0.0706 | Auto-ARIMA | 0.0009 | 0.0015 | Auto-ARIMA |
| Kuwait | 0.0439 | 0.2839 | Auto-ARIMA | 0.0036 | 0.0044 | Auto-ARIMA |
| Kyrgyzstan | 0.1008 | 0.116 | Auto-ARIMA | 0.0023 | 0.0019 | Prophet |
| Laos | 0.1057 | 0.0685 | Prophet | 0.0012 | 0.0028 | Auto-ARIMA |
| Latvia | 0.0955 | 0.017 | Prophet | 0.0006 | 0.0022 | Auto-ARIMA |
| Lebanon | 0.1748 | 0.5272 | Auto-ARIMA | 0.0005 | 0.0057 | Auto-ARIMA |
| Lesotho | 0.0393 | 0.0331 | Prophet | 0.0016 | 0.0042 | Auto-ARIMA |
| Liberia | 0.1677 | 0.0988 | Prophet | 0.0012 | 0.0046 | Auto-ARIMA |
| Libya | 0.666 | 0.7742 | Auto-ARIMA | 0.002 | 0.0121 | Auto-ARIMA |
| Lithuania | 0.0553 | 0.0894 | Auto-ARIMA | 0.0025 | 0.0026 | Auto-ARIMA |
| Luxembourg | 0.0743 | 0.0884 | Auto-ARIMA | 0.0009 | 0.0027 | Auto-ARIMA |
| Madagascar | 0.052 | 0.0077 | Prophet | 0.0026 | 0.0018 | Prophet |
| Malawi | 0.1279 | 0.1088 | Prophet | 0.0023 | 0.0022 | Prophet |
| Malaysia | 0.1716 | 0.0927 | Prophet | 0.0019 | 0.001 | Prophet |
| Maldives | 0.0207 | 0.0138 | Prophet | 0.003 | 0.0043 | Auto-ARIMA |
| Mali | 0.1996 | 0.1743 | Prophet | 0.001 | 0.0004 | Prophet |
| Malta | 0.0534 | 0.2218 | Auto-ARIMA | 0.0016 | 0.0022 | Auto-ARIMA |
| Marshall Is. | 0.2417 | 0.4178 | Auto-ARIMA | 0.0008 | 0.0008 | Auto-ARIMA |
| Mauritania | 0.1937 | 0.1599 | Prophet | 0.0005 | 0.0016 | Auto-ARIMA |
| Mauritius | 0.7175 | 0.7656 | Auto-ARIMA | 0.0026 | 0.0027 | Auto-ARIMA |
| Mexico | 0.068 | 0.1533 | Auto-ARIMA | 0.0012 | 0.0038 | Auto-ARIMA |
| Micronesia | 0.2767 | 1.2286 | Auto-ARIMA | 0.0007 | 0.0003 | Prophet |
| Moldova | 0.0759 | 0.0579 | Prophet | 0.0018 | 0.0017 | Prophet |
| Monaco | 0.0183 | 0.062 | Auto-ARIMA | 4.00E-05 | 0.0002 | Auto-ARIMA |
| Mongolia | 0.3519 | 0.2459 | Prophet | 0.0008 | 0.0007 | Prophet |
| Montenegro | 0.041 | 0.0783 | Auto-ARIMA | 0.0019 | 0.002 | Auto-ARIMA |
| Morocco | 0.2411 | 0.871 | Auto-ARIMA | 0.0011 | 0.0009 | Prophet |
| Mozambique | 0.0032 | 0.0452 | Auto-ARIMA | 0.0019 | 0.0022 | Auto-ARIMA |
| Myanmar | 0.0507 | 0.0847 | Auto-ARIMA | 0.0022 | 0.0032 | Auto-ARIMA |
| N. Mariana Is. | 0.0976 | 0.7454 | Auto-ARIMA | 0.0018 | 0.0015 | Prophet |
| Namibia | 0.0351 | 0.0204 | Prophet | 0.0012 | 0.0065 | Auto-ARIMA |
| Nauru | 0.0187 | 0.2678 | Auto-ARIMA | 0.0017 | 0.0054 | Auto-ARIMA |
| Nepal | 0.1085 | 0.3277 | Auto-ARIMA | 0.002 | 0.0024 | Auto-ARIMA |
| Netherlands | 0.0092 | 0.0177 | Auto-ARIMA | 0.0014 | 0.0007 | Prophet |
| New Zealand | 0.0086 | 0.0854 | Auto-ARIMA | 0.0019 | 0.0028 | Auto-ARIMA |
| Nicaragua | 0.0182 | 0.0532 | Auto-ARIMA | 0.0002 | 0.0033 | Auto-ARIMA |
| Niger | 0.1107 | 0.0899 | Prophet | 0.0007 | 0.0001 | Prophet |
| Nigeria | 0.0681 | 0.1133 | Auto-ARIMA | 0.0011 | 0.0013 | Auto-ARIMA |
| Niue | 0.2809 | 0.1956 | Prophet | 0.0009 | 0.0005 | Prophet |
| North Macedonia | 0.0245 | 0.0354 | Auto-ARIMA | 0.0017 | 0.0031 | Auto-ARIMA |
| Norway | 0.0806 | 0.1465 | Auto-ARIMA | 0.0015 | 0.0028 | Auto-ARIMA |
| Oman | 0.4699 | 0.6742 | Auto-ARIMA | 0.0014 | 0.0009 | Prophet |
| Pakistan | 0.0576 | 0.0583 | Auto-ARIMA | 0.0001 | 0.0006 | Auto-ARIMA |
| Palau | 0.1905 | 0.0864 | Prophet | 0.0011 | 0.0022 | Auto-ARIMA |
| Palestine | 0.1939 | 0.0483 | Prophet | 0.0011 | 0.0052 | Auto-ARIMA |
| Panama | 0.1134 | 0.0864 | Prophet | 0.0057 | 0.0044 | Prophet |
| Papua New Guinea | 0.0562 | 0.128 | Auto-ARIMA | 0.0007 | 0.0023 | Auto-ARIMA |
| Paraguay | 0.3628 | 0.2252 | Prophet | 0.0019 | 0.0029 | Auto-ARIMA |
| Peru | 0.0149 | 0.0288 | Auto-ARIMA | 0.0015 | 0.0031 | Auto-ARIMA |
| Philippines | 0.269 | 0.2866 | Auto-ARIMA | 0.0027 | 0.0025 | Prophet |
| Poland | 0.0557 | 0.1023 | Auto-ARIMA | 0.0029 | 0.0012 | Prophet |
| Portugal | 0.1866 | 0.2897 | Auto-ARIMA | 0.0015 | 0.0009 | Prophet |
| Puerto Rico | 0.3956 | 0.1469 | Prophet | 0.004 | 0.0065 | Auto-ARIMA |
| Qatar | 0.2657 | 0.0445 | Prophet | 0.0006 | 0.001 | Auto-ARIMA |
| Republic of Korea | 0.0393 | 0.0814 | Auto-ARIMA | 0.0013 | 0.0024 | Auto-ARIMA |
| Republic of the Congo | 0.1478 | 0.0595 | Prophet | 0.0011 | 0.0048 | Auto-ARIMA |
| Romania | 0.2161 | 0.1552 | Prophet | 0.0014 | 0.0017 | Auto-ARIMA |
| Russia | 0.0976 | 0.082 | Prophet | 0.002 | 0.005 | Auto-ARIMA |
| Rwanda | 0.0237 | 0.0656 | Auto-ARIMA | 0.0033 | 0.003 | Prophet |
| S. Sudan | 0.0148 | 0.0826 | Auto-ARIMA | 0.0009 | 0.0033 | Auto-ARIMA |
| Saint Kitts and Nevis | 0.0426 | 0.0164 | Prophet | 0.0012 | 0.001 | Prophet |
| Saint Lucia | 0.0272 | 0.0943 | Auto-ARIMA | 0.0014 | 0.001 | Prophet |
| Samoa | 0.015 | 0.0379 | Auto-ARIMA | 0.0006 | 0.0014 | Auto-ARIMA |
| San Marino | 0.0204 | 0.0232 | Auto-ARIMA | 0.0004 | 0.0022 | Auto-ARIMA |
| Sao Tome and Principe | 0.12 | 0.0736 | Prophet | 0.0012 | 0.0021 | Auto-ARIMA |
| Saudi Arabia | 0.2812 | 0.3809 | Auto-ARIMA | 0.0012 | 0.0042 | Auto-ARIMA |
| Senegal | 0.5029 | 0.6262 | Auto-ARIMA | 0.0017 | 0.0006 | Prophet |
| Serbia | 0.0074 | 0.0611 | Auto-ARIMA | 0.0025 | 0.0005 | Prophet |
| Seychelles | 0.0505 | 0.1304 | Auto-ARIMA | 0.0027 | 0.0008 | Prophet |
| Sierra Leone | 0.0304 | 0.0196 | Prophet | 0.0016 | 0.0012 | Prophet |
| Singapore | 0.1024 | 0.1095 | Auto-ARIMA | 0.0001 | 0.0008 | Auto-ARIMA |
| Slovakia | 0.2166 | 0.1281 | Prophet | 0.0002 | 0.0026 | Auto-ARIMA |
| Slovenia | 0.0428 | 0.0777 | Auto-ARIMA | 0.0012 | 0.0012 | Prophet |
| Solomon Is. | 0.0181 | 0.1709 | Auto-ARIMA | 0.0004 | 0.0015 | Auto-ARIMA |
| Somalia | 0.0471 | 0.0142 | Prophet | 3.00E-05 | 0.0003 | Auto-ARIMA |
| South Africa | 0.0424 | 0.0182 | Prophet | 0.002 | 0.0025 | Auto-ARIMA |
| Spain | 0.3789 | 0.5219 | Auto-ARIMA | 0.0013 | 0.0005 | Prophet |
| Sri Lanka | 0.0482 | 0.0679 | Auto-ARIMA | 0.0021 | 0.0047 | Auto-ARIMA |
| St. Vin. and Gren. | 0.1166 | 0.0516 | Prophet | 0.0011 | 0.0021 | Auto-ARIMA |
| Sudan | 0.1852 | 0.1709 | Prophet | 0.0005 | 0.0034 | Auto-ARIMA |
| Suriname | 0.0116 | 0.251 | Auto-ARIMA | 0.0011 | 0.0003 | Prophet |
| Sweden | 0.0525 | 0.1055 | Auto-ARIMA | 0.0019 | 0.0004 | Prophet |
| Switzerland | 0.0033 | 0.1772 | Auto-ARIMA | 0.0015 | 0.0013 | Prophet |
| Syria | 0.2689 | 0.1263 | Prophet | 0.0009 | 0.0089 | Auto-ARIMA |
| Tajikistan | 0.0736 | 0.0315 | Prophet | 0.0012 | 0.0005 | Prophet |
| Tanzania | 0.018 | 0.0474 | Auto-ARIMA | 0.0017 | 0.0005 | Prophet |
| Thailand | 0.1002 | 0.0694 | Prophet | 0.0018 | 0.0028 | Auto-ARIMA |
| Timor-Leste | 0.056 | 0.0535 | Prophet | 0.0019 | 0.005 | Auto-ARIMA |
| Togo | 0.1069 | 0.1148 | Auto-ARIMA | 0.002 | 0.0008 | Prophet |
| Tonga | 0.1145 | 0.3186 | Auto-ARIMA | 0.001 | 0.0006 | Prophet |
| Trinidad and Tobago | 0.0157 | 0.0539 | Auto-ARIMA | 0.001 | 0.0025 | Auto-ARIMA |
| Tunisia | 0.129 | 0.111 | Prophet | 0.0005 | 0.0007 | Auto-ARIMA |
| Turkey | 0.0595 | 0.7116 | Auto-ARIMA | 0.0017 | 0.0036 | Auto-ARIMA |
| Turkmenistan | 0.0345 | 0.0356 | Auto-ARIMA | 0.0013 | 0.0031 | Auto-ARIMA |
| Tuvalu | 0.0832 | 0.0796 | Prophet | 0.0013 | 0.0013 | Auto-ARIMA |
| U.S. Virgin Is. | 0.3413 | 0.15 | Prophet | 0.0034 | 0.0001 | Prophet |
| Uganda | 0.067 | 0.0912 | Auto-ARIMA | 0.0032 | 0.0019 | Prophet |
| Ukraine | 0.0651 | 0.1008 | Auto-ARIMA | 0.0032 | 0.0036 | Auto-ARIMA |
| United Arab Emirates | 0.3244 | 0.009 | Prophet | 1.00E-04 | 0.0047 | Auto-ARIMA |
| United Kingdom | 0.1958 | 0.379 | Auto-ARIMA | 0.0033 | 0.0015 | Prophet |
| United States | 0.073 | 0.0801 | Auto-ARIMA | 0.0023 | 0.0026 | Auto-ARIMA |
| Uruguay | 0.0437 | 0.0492 | Auto-ARIMA | 0.0005 | 0.0086 | Auto-ARIMA |
| Uzbekistan | 0.0715 | 0.1374 | Auto-ARIMA | 0.0014 | 0.0017 | Auto-ARIMA |
| Vanuatu | 0.1827 | 0.0345 | Prophet | 0.0011 | 0.0005 | Prophet |
| Venezuela | 0.0846 | 0.1093 | Auto-ARIMA | 0.0011 | 0.0015 | Auto-ARIMA |
| Vietnam | 0.1596 | 0.4115 | Auto-ARIMA | 0.002 | 0.0012 | Prophet |
| Yemen | 0.0734 | 0.1636 | Auto-ARIMA | 0.0003 | 0.0031 | Auto-ARIMA |
| Zambia | 0.161 | 0.0691 | Prophet | 0.0011 | 0.0023 | Auto-ARIMA |
| Zimbabwe | 0.0697 | 0.0366 | Prophet | 0.0017 | 0.0063 | Auto-ARIMA |

- 1. Supplementary Table S5. High Fasting Plasma Glucose (HFPG) exposure rates, socio-demographic index (SDI) in 1990–2040 in 204 countries and territories.

| **Country (**Territory) | **High Fasting Plasma Glucose** | | | **Socio-Demographic Index** | | |
| --- | --- | --- | --- | --- | --- | --- |
|  | **1990** | **2021** | **2040** | **1990** | **2021** | **2040** |
| Afghanistan | 12.94 | 21.77 | 27.67 | 0.17 | 0.34 | 0.48 |
| Albania | 6.13 | 9.70 | 12.9 | 0.56 | 0.71 | 0.76 |
| Algeria | 12.64 | 22.41 | 29.62 | 0.46 | 0.66 | 0.78 |
| American Samoa | 37.82 | 46.80 | 51.93 | 0.61 | 0.72 | 0.77 |
| Andorra | 9.65 | 14.22 | 18.21 | 0.76 | 0.87 | 0.92 |
| Angola | 8.27 | 11.76 | 14.95 | 0.27 | 0.45 | 0.57 |
| Antigua and Barb. | 14.08 | 19.82 | 23.4 | 0.61 | 0.75 | 0.82 |
| Argentina | 8.78 | 15.20 | 18.72 | 0.59 | 0.72 | 0.81 |
| Armenia | 7.18 | 11.29 | 13.95 | 0.54 | 0.7 | 0.78 |
| Australia | 8.58 | 14.90 | 17.87 | 0.73 | 0.84 | 0.91 |
| Austria | 4.96 | 8.73 | 10.64 | 0.75 | 0.85 | 0.91 |
| Azerbaijan | 6.96 | 12.57 | 15.65 | 0.6 | 0.69 | 0.74 |
| Bahamas | 14.97 | 22.15 | 26.23 | 0.69 | 0.81 | 0.88 |
| Bahrain | 16.29 | 25.93 | 30.78 | 0.58 | 0.75 | 0.86 |
| Bangladesh | 10.23 | 17.82 | 21.71 | 0.23 | 0.49 | 0.68 |
| Barbados | 19.11 | 23.77 | 27.8 | 0.65 | 0.75 | 0.79 |
| Belarus | 5.39 | 7.42 | 8.83 | 0.62 | 0.78 | 0.88 |
| Belgium | 8.49 | 12.92 | 16.61 | 0.74 | 0.85 | 0.91 |
| Belize | 10.96 | 17.49 | 19.39 | 0.42 | 0.61 | 0.68 |
| Benin | 5.43 | 9.49 | 12.35 | 0.22 | 0.37 | 0.51 |
| Bermuda | 13.15 | 20.47 | 25.96 | 0.7 | 0.82 | 0.86 |
| Bhutan | 9.20 | 12.99 | 15.6 | 0.22 | 0.47 | 0.56 |
| Bolivia | 8.40 | 13.85 | 17.72 | 0.42 | 0.6 | 0.7 |
| Bosnia and Herz. | 8.15 | 15.41 | 18.09 | 0.54 | 0.72 | 0.8 |
| Botswana | 6.27 | 10.21 | 12.65 | 0.42 | 0.64 | 0.71 |
| Brazil | 12.47 | 18.75 | 22.33 | 0.5 | 0.65 | 0.73 |
| Brunei | 16.69 | 22.38 | 25.75 | 0.67 | 0.81 | 0.86 |
| Bulgaria | 9.65 | 13.35 | 15.62 | 0.63 | 0.77 | 0.86 |
| Burkina Faso | 5.49 | 8.78 | 12.18 | 0.13 | 0.29 | 0.4 |
| Burundi | 5.29 | 6.73 | 8.26 | 0.21 | 0.29 | 0.34 |
| Côte d'Ivoire | 7.34 | 11.80 | 14.99 | 0.28 | 0.43 | 0.52 |
| Cabo Verde | 10.49 | 18.91 | 24.91 | 0.28 | 0.53 | 0.66 |
| Cambodia | 4.59 | 7.88 | 10.26 | 0.29 | 0.47 | 0.59 |
| Cameroon | 7.06 | 13.02 | 17.41 | 0.3 | 0.48 | 0.64 |
| Canada | 5.61 | 10.85 | 16.18 | 0.78 | 0.87 | 0.92 |
| Central African Rep. | 14.27 | 18.88 | 21.17 | 0.22 | 0.31 | 0.37 |
| Chad | 7.47 | 11.08 | 13.37 | 0.11 | 0.24 | 0.32 |
| Chile | 8.61 | 15.92 | 18.6 | 0.59 | 0.77 | 0.87 |
| China | 12.16 | 17.23 | 15.42 | 0.46 | 0.72 | 0.88 |
| Colombia | 10.14 | 13.85 | 19.32 | 0.48 | 0.66 | 0.73 |
| Comoros | 5.39 | 7.53 | 9.17 | 0.27 | 0.48 | 0.55 |
| Cook Is. | 34.43 | 47.71 | 55.54 | 0.56 | 0.78 | 0.89 |
| Costa Rica | 11.90 | 18.92 | 22.25 | 0.53 | 0.7 | 0.83 |
| Croatia | 8.86 | 13.90 | 16.7 | 0.67 | 0.8 | 0.87 |
| Cuba | 14.49 | 22.19 | 27.72 | 0.56 | 0.67 | 0.74 |
| Cyprus | 10.58 | 13.91 | 17.08 | 0.65 | 0.84 | 0.9 |
| Czechia | 9.06 | 15.53 | 19.6 | 0.68 | 0.83 | 0.88 |
| Dem. Rep. Korea | 10.50 | 13.91 | 16.21 | 0.5 | 0.57 | 0.61 |
| Democratic Republic of the Congo | 17.44 | 19.27 | 23.14 | 0.29 | 0.38 | 0.47 |
| Denmark | 6.23 | 10.47 | 13.08 | 0.8 | 0.9 | 0.96 |
| Djibouti | 4.75 | 7.30 | 9.49 | 0.34 | 0.49 | 0.62 |
| Dominica | 15.62 | 25.36 | 28.76 | 0.56 | 0.75 | 0.81 |
| Dominican Rep. | 7.88 | 14.57 | 19.19 | 0.44 | 0.62 | 0.71 |
| Ecuador | 10.20 | 16.99 | 21.86 | 0.52 | 0.66 | 0.75 |
| Egypt | 5.83 | 15.15 | 23.26 | 0.42 | 0.61 | 0.73 |
| El Salvador | 9.86 | 17.37 | 21.13 | 0.37 | 0.56 | 0.68 |
| Eq. Guinea | 11.12 | 29.57 | 41.22 | 0.27 | 0.66 | 0.77 |
| Eritrea | 4.30 | 6.38 | 7.94 | 0.22 | 0.4 | 0.49 |
| Estonia | 6.54 | 10.82 | 12.48 | 0.67 | 0.84 | 0.9 |
| eSwatini | 6.86 | 11.73 | 12.73 | 0.4 | 0.59 | 0.63 |
| Ethiopia | 6.63 | 6.52 | 6.34 | 0.15 | 0.36 | 0.44 |
| Fiji | 24.17 | 36.09 | 43.33 | 0.53 | 0.68 | 0.75 |
| Finland | 12.36 | 18.61 | 21.44 | 0.76 | 0.86 | 0.92 |
| France | 7.81 | 11.35 | 14.19 | 0.73 | 0.84 | 0.89 |
| Gabon | 13.27 | 27.06 | 36.93 | 0.46 | 0.63 | 0.78 |
| Gambia | 6.57 | 11.00 | 14.7 | 0.24 | 0.41 | 0.52 |
| Georgia | 5.39 | 10.31 | 8.71 | 0.66 | 0.73 | 0.81 |
| Germany | 9.40 | 13.85 | 18.91 | 0.82 | 0.9 | 0.95 |
| Ghana | 6.57 | 13.09 | 17.06 | 0.37 | 0.56 | 0.71 |
| Greece | 8.86 | 12.28 | 14.18 | 0.67 | 0.79 | 0.84 |
| Greenland | 4.14 | 8.86 | 20.74 | 0.73 | 0.83 | 0.84 |
| Grenada | 14.56 | 22.37 | 26.56 | 0.44 | 0.67 | 0.77 |
| Guam | 23.70 | 36.10 | 43.22 | 0.68 | 0.8 | 0.87 |
| Guatemala | 10.54 | 18.29 | 20.71 | 0.31 | 0.54 | 0.67 |
| Guinea | 6.83 | 10.47 | 15.84 | 0.18 | 0.34 | 0.48 |
| Guinea-Bissau | 7.44 | 11.12 | 13.41 | 0.21 | 0.35 | 0.47 |
| Guyana | 19.08 | 28.32 | 34.22 | 0.46 | 0.65 | 0.77 |
| Haiti | 12.87 | 16.54 | 18.38 | 0.31 | 0.45 | 0.48 |
| Honduras | 10.34 | 15.85 | 18.28 | 0.33 | 0.51 | 0.6 |
| Hungary | 9.40 | 14.14 | 15 | 0.65 | 0.79 | 0.87 |
| Iceland | 9.31 | 14.96 | 19.17 | 0.76 | 0.88 | 0.95 |
| India | 11.79 | 18.02 | 22.44 | 0.33 | 0.58 | 0.71 |
| Indonesia | 6.70 | 10.52 | 13.17 | 0.46 | 0.66 | 0.78 |
| Iran | 9.03 | 16.96 | 21.84 | 0.45 | 0.7 | 0.81 |
| Iraq | 21.63 | 37.17 | 46.45 | 0.41 | 0.66 | 0.84 |
| Ireland | 9.30 | 13.88 | 17.6 | 0.72 | 0.87 | 0.9 |
| Israel | 10.37 | 16.73 | 20.49 | 0.71 | 0.81 | 0.88 |
| Italy | 9.02 | 11.85 | 13.59 | 0.71 | 0.81 | 0.86 |
| Jamaica | 8.09 | 11.71 | 14.33 | 0.53 | 0.68 | 0.75 |
| Japan | 12.39 | 13.50 | 12.19 | 0.79 | 0.87 | 0.92 |
| Jordan | 16.04 | 26.40 | 32.82 | 0.54 | 0.73 | 0.83 |
| Kazakhstan | 6.94 | 12.42 | 15.32 | 0.59 | 0.73 | 0.79 |
| Kenya | 5.69 | 8.21 | 10.31 | 0.33 | 0.52 | 0.69 |
| Kiribati | 30.31 | 37.59 | 41.78 | 0.41 | 0.53 | 0.6 |
| Kuwait | 17.39 | 25.47 | 30.77 | 0.66 | 0.85 | 0.89 |
| Kyrgyzstan | 5.76 | 9.48 | 13.24 | 0.52 | 0.6 | 0.7 |
| Laos | 8.17 | 11.23 | 13.42 | 0.26 | 0.49 | 0.58 |
| Latvia | 6.55 | 10.76 | 12.93 | 0.68 | 0.83 | 0.92 |
| Lebanon | 13.06 | 21.79 | 27.13 | 0.54 | 0.74 | 0.78 |
| Lesotho | 4.60 | 9.16 | 11.48 | 0.34 | 0.51 | 0.61 |
| Liberia | 9.44 | 15.85 | 20.06 | 0.24 | 0.35 | 0.43 |
| Libya | 10.52 | 18.69 | 11.94 | 0.53 | 0.73 | 0.83 |
| Lithuania | 5.88 | 8.86 | 10.83 | 0.67 | 0.86 | 0.91 |
| Luxembourg | 7.82 | 16.19 | 21.09 | 0.78 | 0.88 | 0.92 |
| Madagascar | 4.85 | 6.63 | 8.33 | 0.28 | 0.4 | 0.56 |
| Malawi | 4.43 | 6.39 | 8.42 | 0.2 | 0.38 | 0.54 |
| Malaysia | 13.51 | 20.14 | 26.08 | 0.55 | 0.74 | 0.84 |
| Maldives | 8.72 | 11.59 | 14.37 | 0.33 | 0.65 | 0.7 |
| Mali | 14.66 | 19.51 | 22.89 | 0.13 | 0.27 | 0.39 |
| Malta | 8.74 | 14.79 | 20.17 | 0.66 | 0.8 | 0.87 |
| Marshall Is. | 40.94 | 50.79 | 56.72 | 0.43 | 0.57 | 0.67 |
| Mauritania | 5.80 | 8.79 | 10.99 | 0.34 | 0.5 | 0.64 |
| Mauritius | 19.36 | 27.93 | 24.74 | 0.54 | 0.72 | 0.82 |
| Mexico | 21.76 | 26.45 | 29.28 | 0.5 | 0.66 | 0.76 |
| Micronesia | 25.01 | 31.58 | 35.88 | 0.46 | 0.59 | 0.65 |
| Moldova | 7.97 | 11.92 | 13.81 | 0.6 | 0.73 | 0.84 |
| Monaco | 10.25 | 15.63 | 19.62 | 0.85 | 0.91 | 0.94 |
| Mongolia | 5.43 | 9.93 | 14.99 | 0.47 | 0.62 | 0.69 |
| Montenegro | 10.86 | 16.19 | 20.11 | 0.67 | 0.8 | 0.85 |
| Morocco | 14.23 | 32.34 | 43.36 | 0.36 | 0.56 | 0.71 |
| Mozambique | 4.22 | 6.83 | 8.52 | 0.17 | 0.33 | 0.48 |
| Myanmar | 10.31 | 14.89 | 18.42 | 0.32 | 0.53 | 0.57 |
| N. Mariana Is. | 35.62 | 40.12 | 46 | 0.71 | 0.77 | 0.86 |
| Namibia | 8.30 | 11.84 | 14.21 | 0.45 | 0.62 | 0.71 |
| Nauru | 23.77 | 28.99 | 35.9 | 0.54 | 0.63 | 0.74 |
| Nepal | 8.51 | 15.14 | 27.87 | 0.2 | 0.43 | 0.55 |
| Netherlands | 8.39 | 12.37 | 15.66 | 0.79 | 0.89 | 0.94 |
| New Zealand | 11.05 | 18.28 | 22.67 | 0.75 | 0.85 | 0.91 |
| Nicaragua | 10.62 | 15.17 | 17.98 | 0.35 | 0.52 | 0.59 |
| Niger | 10.32 | 13.55 | 15.92 | 0.08 | 0.17 | 0.24 |
| Nigeria | 6.39 | 9.45 | 11.46 | 0.31 | 0.5 | 0.64 |
| Niue | 27.86 | 41.23 | 48.56 | 0.59 | 0.73 | 0.8 |
| North Macedonia | 9.53 | 15.66 | 17.8 | 0.61 | 0.75 | 0.83 |
| Norway | 12.54 | 15.22 | 18.33 | 0.8 | 0.92 | 0.94 |
| Oman | 10.68 | 21.72 | 35.8 | 0.43 | 0.77 | 0.87 |
| Pakistan | 12.45 | 19.87 | 20.64 | 0.31 | 0.5 | 0.62 |
| Palau | 29.21 | 37.93 | 42.61 | 0.66 | 0.75 | 0.77 |
| Palestine | 11.78 | 18.74 | 23.95 | 0.4 | 0.63 | 0.77 |
| Panama | 10.47 | 17.17 | 22.43 | 0.55 | 0.71 | 0.88 |
| Papua New Guinea | 16.97 | 21.88 | 24.14 | 0.31 | 0.42 | 0.48 |
| Paraguay | 9.38 | 16.26 | 21.44 | 0.47 | 0.64 | 0.73 |
| Peru | 5.77 | 9.35 | 13.09 | 0.51 | 0.66 | 0.73 |
| Philippines | 7.68 | 9.19 | 12.26 | 0.51 | 0.65 | 0.8 |
| Poland | 11.14 | 16.58 | 20.13 | 0.63 | 0.81 | 0.89 |
| Portugal | 11.32 | 17.01 | 20.55 | 0.6 | 0.74 | 0.8 |
| Puerto Rico | 20.95 | 32.81 | 37.09 | 0.66 | 0.83 | 0.92 |
| Qatar | 15.39 | 26.29 | 34.69 | 0.65 | 0.85 | 0.94 |
| Republic of Korea | 11.48 | 16.02 | 22.59 | 0.69 | 0.89 | 0.95 |
| Republic of the Congo | 8.22 | 13.06 | 16.55 | 0.42 | 0.58 | 0.67 |
| Romania | 7.63 | 13.48 | 17.21 | 0.62 | 0.77 | 0.86 |
| Russia | 6.59 | 10.62 | 12.23 | 0.67 | 0.81 | 0.89 |
| Rwanda | 4.15 | 5.14 | 6.51 | 0.28 | 0.44 | 0.59 |
| S. Sudan | 5.08 | 7.32 | 8.93 | 0.21 | 0.28 | 0.31 |
| Saint Kitts and Nevis | 15.26 | 21.61 | 26.8 | 0.58 | 0.75 | 0.83 |
| Saint Lucia | 16.22 | 23.88 | 28.32 | 0.5 | 0.67 | 0.74 |
| Samoa | 26.53 | 39.49 | 47.48 | 0.49 | 0.59 | 0.66 |
| San Marino | 9.77 | 14.61 | 17.28 | 0.81 | 0.89 | 0.91 |
| Sao Tome and Principe | 5.75 | 11.27 | 17.12 | 0.31 | 0.51 | 0.61 |
| Saudi Arabia | 16.01 | 29.93 | 37.29 | 0.54 | 0.82 | 0.9 |
| Senegal | 10.42 | 14.68 | 9.52 | 0.24 | 0.41 | 0.55 |
| Serbia | 9.70 | 14.60 | 17.49 | 0.63 | 0.79 | 0.87 |
| Seychelles | 15.22 | 24.83 | 33.19 | 0.58 | 0.73 | 0.83 |
| Sierra Leone | 6.49 | 10.13 | 12.64 | 0.21 | 0.36 | 0.49 |
| Singapore | 17.62 | 19.82 | 19.72 | 0.69 | 0.86 | 0.89 |
| Slovakia | 8.33 | 12.20 | 14.44 | 0.65 | 0.81 | 0.86 |
| Slovenia | 8.72 | 13.13 | 16.61 | 0.73 | 0.84 | 0.88 |
| Solomon Is. | 14.10 | 18.49 | 20.37 | 0.3 | 0.43 | 0.5 |
| Somalia | 5.24 | 7.07 | 8.62 | 0.05 | 0.08 | 0.1 |
| South Africa | 7.92 | 12.36 | 14.47 | 0.54 | 0.68 | 0.72 |
| Spain | 11.02 | 15.34 | 19.48 | 0.64 | 0.77 | 0.82 |
| Sri Lanka | 9.27 | 15.76 | 18.75 | 0.52 | 0.7 | 0.81 |
| St. Vin. and Gren. | 14.88 | 22.02 | 25.66 | 0.48 | 0.64 | 0.74 |
| Sudan | 9.46 | 16.64 | 20.48 | 0.29 | 0.54 | 0.7 |
| Suriname | 18.80 | 28.39 | 33.83 | 0.5 | 0.63 | 0.69 |
| Sweden | 9.47 | 14.23 | 17.29 | 0.79 | 0.89 | 0.94 |
| Switzerland | 10.40 | 14.61 | 17.3 | 0.86 | 0.93 | 0.97 |
| Syria | 11.08 | 17.86 | 21.22 | 0.43 | 0.62 | 0.74 |
| Tajikistan | 8.45 | 12.59 | 15.52 | 0.47 | 0.54 | 0.63 |
| Tanzania | 4.52 | 7.24 | 9.26 | 0.26 | 0.45 | 0.61 |
| Thailand | 8.63 | 12.46 | 15.63 | 0.51 | 0.68 | 0.75 |
| Timor-Leste | 5.00 | 8.65 | 11.24 | 0.26 | 0.44 | 0.49 |
| Togo | 5.23 | 7.75 | 11.6 | 0.27 | 0.41 | 0.55 |
| Tonga | 22.24 | 33.50 | 40.97 | 0.49 | 0.63 | 0.73 |
| Trinidad and Tobago | 16.52 | 21.76 | 24.86 | 0.62 | 0.77 | 0.81 |
| Tunisia | 10.52 | 19.35 | 23.82 | 0.47 | 0.68 | 0.77 |
| Turkey | 8.77 | 17.63 | 21.83 | 0.46 | 0.71 | 0.84 |
| Turkmenistan | 6.31 | 12.39 | 17.45 | 0.56 | 0.68 | 0.75 |
| Tuvalu | 16.29 | 20.22 | 22.85 | 0.41 | 0.58 | 0.67 |
| U.S. Virgin Is. | 18.79 | 31.02 | 32.84 | 0.66 | 0.82 | 0.87 |
| Uganda | 5.37 | 8.16 | 9.88 | 0.19 | 0.42 | 0.59 |
| Ukraine | 6.89 | 9.18 | 10.72 | 0.65 | 0.76 | 0.82 |
| United Arab Emirates | 16.72 | 29.64 | 35.11 | 0.64 | 0.85 | 0.91 |
| United Kingdom | 7.12 | 12.51 | 15.64 | 0.74 | 0.86 | 0.93 |
| United States | 12.62 | 24.57 | 29.99 | 0.76 | 0.86 | 0.92 |
| Uruguay | 6.60 | 12.31 | 16.43 | 0.58 | 0.72 | 0.8 |
| Uzbekistan | 5.94 | 12.69 | 15.44 | 0.5 | 0.66 | 0.76 |
| Vanuatu | 19.55 | 29.33 | 34.25 | 0.35 | 0.47 | 0.55 |
| Venezuela | 10.73 | 15.63 | 18.51 | 0.52 | 0.6 | 0.58 |
| Vietnam | 5.90 | 7.84 | 12.65 | 0.41 | 0.63 | 0.74 |
| Yemen | 7.86 | 11.84 | 13.31 | 0.22 | 0.45 | 0.52 |
| Zambia | 7.91 | 11.86 | 15.14 | 0.3 | 0.51 | 0.59 |
| Zimbabwe | 7.17 | 10.65 | 11.61 | 0.4 | 0.47 | 0.47 |

- 1. Supplementary Table S6. Age-standardized mortality rates and average annual percentage changes for 1990–2040 and differences between 1990–2021 vs. 2022–2040 periods of type 2 diabetes mellitus in 204 countries and territories.

| **Country**  **(Territory)** | **1990 ASMR** | **2021 ASMR** | **2040 ASMR** | **1990–2021 ASMR** | **2022–2040 ASMR** | **Gap between the two periods** | **1990–2021 AAPC** | **2022–2040 AAPC** |
| --- | --- | --- | --- | --- | --- | --- | --- | --- |
| Afghanistan | 24.43 (18.87–32.85) | 39.34 (30.12–51.43) | 47.37 (22.56–72.18) | 30.86 | 43.55 | 12.69 | 1.55 (1.48–1.62) | 0.947 (0.943–0.95) |
| Albania | 4.8 (3.96–5.76) | 4.67 (3.73–5.93) | 4.09 (-7.2–15.39) | 4.56 | 4.31 | -0.24 | -0.18 (-0.81–0.45) | -0.602 (-0.612–-0.592) |
| Algeria | 12.24 (9.93–15.86) | 18.54 (15.02–22.59) | 24.5 (5.55–43.45) | 14.77 | 21.8 | 7.02 | 1.37 (1.12–1.63) | 1.392 (1.39–1.395) |
| American Samoa | 93.06 (78.57–106.45) | 112.78 (96.7–133.39) | 78.93 (-65.22–223.07) | 115.14 | 93.76 | -21.37 | 0.77 (0.34–1.21) | -1.915 (-1.924–-1.907) |
| Andorra | 12.16 (9.19–16.06) | 8.29 (6.03–10.84) | 2.66 (-4.17–9.49) | 10.49 | 4.85 | -5.64 | -1.38 (-1.66–-1.09) | -5.783 (-5.787–-5.779) |
| Angola | 48.18 (38.93–60.2) | 48.2 (37.76–59.42) | 53.29 (-28.35–134.93) | 46.69 | 50.38 | 3.69 | 0.03 (-0.24–0.3) | 0.579 (0.565–0.594) |
| Antigua and Barb. | 64.99 (59.98–69.8) | 57.21 (52.58–60.79) | 23.21 (-9.79–56.21) | 68.72 | 36.34 | -32.38 | -0.51 (-1.28–0.26) | -4.516 (-4.517–-4.515) |
| Argentina | 22.64 (21.26–23.75) | 15.97 (14.84–16.95) | 6.23 (-4.42–16.87) | 21.6 | 10.27 | -11.34 | -0.91 (-1.85–0.03) | -4.94 (-4.944–-4.935) |
| Armenia | 17.3 (15.82–18.76) | 14.12 (12.51–16.02) | 11.08 (-48.56–70.71) | 27.77 | 12.22 | -15.54 | -0.76 (-2.42–0.93) | -1.356 (-1.382–-1.33) |
| Australia | 11.11 (10.1–11.92) | 8.42 (7.38–9.14) | 8.64 (-14.18–31.47) | 10.57 | 8.38 | -2.19 | -0.83 (-1.52–-0.13) | 0.144 (0.13–0.157) |
| Austria | 14.07 (12.97–14.9) | 9.09 (7.78–9.89) | 3.25 (-13.94–20.44) | 11.89 | 5.66 | -6.23 | -1.33 (-2.37–-0.27) | -5.355 (-5.409–-5.301) |
| Azerbaijan | 10.74 (8.56–13.43) | 16.94 (12.15–21.87) | 7.84 (-13.24–28.93) | 17.63 | 11.94 | -5.69 | 1.47 (1.09–1.85) | -4.131 (-4.155–-4.106) |
| Bahamas | 47.98 (44.23–51.55) | 35.68 (29.29–43.5) | 30.46 (-38.3–99.22) | 45.83 | 32.48 | -13.35 | -1.01 (-2.11–0.10) | -0.821 (-0.834–-0.809) |
| Bahrain | 106.06 (92.68–119.05) | 126.21 (103.72–145.43) | 99.88 (10.54–189.21) | 134.06 | 108.82 | -25.24 | 0.51 (-0.05–1.08) | -1.043 (-1.058–-1.027) |
| Bangladesh | 28.08 (24.02–32.1) | 35.06 (28.43–42.53) | 35.58 (-16.55–87.71) | 35.36 | 34.92 | -0.44 | 0.82 (0.06–1.60) | 0.103 (0.086–0.12) |
| Barbados | 74.28 (69.82–78.76) | 59.37 (46.96–72.01) | 30.75 (-43.34–104.85) | 71.11 | 42.51 | -28.59 | -0.62 (-1.07–-0.17) | -3.367 (-3.403–-3.331) |
| Belarus | 3.74 (3.5–4.02) | 4.01 (3.29–4.73) | 4.56 (-19.01–28.13) | 3.48 | 4.11 | 0.63 | 0.13 (-1.68–1.97) | 0.632 (0.539–0.725) |
| Belgium | 11.19 (10.1–12.05) | 5.01 (4.27–5.47) | 2.72 (-3.03–8.48) | 8.23 | 3.76 | -4.47 | -2.49 (-3.25–-1.72) | -3.227 (-3.27–-3.185) |
| Belize | 47.26 (44.02–50.19) | 53.99 (47.76–60.78) | 34.62 (-70.45–139.68) | 59.84 | 42.8 | -17.05 | 0.41 (-0.53–1.36) | -2.232 (-2.241–-2.224) |
| Benin | 27.35 (23.1–32.13) | 36 (29.43–43.65) | 37.75 (6–69.49) | 34.08 | 37.1 | 3.02 | 0.9 (0.76–1.03) | 0.199 (0.194–0.205) |
| Bermuda | 35.26 (32.92–37.49) | 16.58 (14.07–20.16) | 15.8 (0.07–31.53) | 23.31 | 16.04 | -7.27 | -2.52 (-3.69–-1.33) | -0.174 (-0.176–-0.172) |
| Bhutan | 23.05 (17.43–29.62) | 31.34 (23.1–39.96) | 32.44 (18.73–46.16) | 26.92 | 32.51 | 5.59 | 1 (0.83–1.18) | 0.071 (0.063–0.078) |
| Bolivia | 39.16 (33.27–47.3) | 42.27 (34.19–54.55) | 39.4 (21.41–57.39) | 41.21 | 41.54 | 0.33 | 0.25 (0.1–0.4) | -0.519 (-0.523–-0.514) |
| Bosnia and Herz. | 14.48 (12.54–16.97) | 30.06 (24.06–37.16) | 10.5 (-9.9–30.9) | 23.69 | 18.09 | -5.6 | 2.48 (1.59–3.37) | -5.292 (-5.296–-5.288) |
| Botswana | 57.25 (44.5–72.92) | 59.85 (49.28–72.97) | 33.17 (-69.29–135.64) | 64.06 | 44.35 | -19.71 | 0.14 (-0.87–1.17) | -2.989 (-2.994–-2.984) |
| Brazil | 31.51 (29.5–32.75) | 25.83 (23.55–27.28) | 21.21 (-2.28–44.7) | 30.04 | 24.1 | -5.94 | -0.59 (-1.15–-0.02) | -1.282 (-1.288–-1.276) |
| Brunei | 81.54 (70.12–93.42) | 49.92 (43.54–57.18) | 26.26 (2.16–50.37) | 69.22 | 36.83 | -32.38 | -1.66 (-2.24–-1.07) | -3.565 (-3.591–-3.54) |
| Bulgaria | 16.17 (14.96–17.45) | 15.59 (13.6–17.65) | 12.31 (-15.75–40.37) | 17.13 | 13.6 | -3.53 | 0.01 (-0.79–0.81) | -1.236 (-1.259–-1.214) |
| Burkina Faso | 34.64 (29.22–41.28) | 33.41 (27.26–40.39) | 27.33 (8.43–46.23) | 34.82 | 30.11 | -4.71 | -0.1 (-0.33–0.13) | -1.077 (-1.082–-1.072) |
| Burundi | 51.84 (39–68.06) | 44.17 (31.07–64.84) | 45.94 (4.83–87.06) | 48.74 | 45.16 | -3.58 | -0.52 (-0.7–-0.34) | 0.192 (0.19–0.194) |
| Cabo Verde | 10.82 (9.13–12.67) | 35.47 (28.77–42.61) | 29.92 (-37.02–96.86) | 24.25 | 32.89 | 8.64 | 3.94 (3.34–4.54) | -0.93 (-0.947–-0.913) |
| Cambodia | 28 (22.77–33.78) | 32.01 (23.23–41.71) | 36.13 (13.93–58.34) | 28.64 | 34.39 | 5.76 | 0.44 (0.34–0.53) | 0.601 (0.598–0.604) |
| Cameroon | 37.99 (30.26–47.09) | 50.33 (38.65–65.54) | 44.5 (17.82–71.18) | 49.3 | 47.34 | -1.96 | 0.92 (0.8–1.04) | -0.661 (-0.68–-0.642) |
| Canada | 12.61 (11.51–13.49) | 7.53 (6.67–8.16) | 5.04 (-5.57–15.65) | 12.34 | 6.07 | -6.26 | -1.55 (-2.18–-0.91) | -2.127 (-2.144–-2.11) |
| Central African Rep. | 61.22 (51.5–70.68) | 61.79 (47.91–77.2) | 52.53 (8.3–96.77) | 63.93 | 56.82 | -7.11 | 0.04 (-0.09–0.18) | -0.877 (-0.883–-0.871) |
| Chad | 22.7 (17.61–29.76) | 35.47 (27.26–46.38) | 36.45 (11.01–61.9) | 32.24 | 36.19 | 3.95 | 1.45 (1.25–1.64) | 0.088 (0.086–0.091) |
| Chile | 15.64 (14.83–16.57) | 10.91 (9.73–11.77) | 7.94 (-33.04–48.92) | 15.86 | 9.28 | -6.58 | -1.28 (-2.43–-0.11) | -1.665 (-1.706–-1.624) |
| China | 9.29 (8.16–10.49) | 8.74 (7.26–10.35) | 7.2 (-0.6–15.01) | 9.92 | 8.05 | -1.88 | -0.15 (-0.39–0.1) | -1.084 (-1.086–-1.081) |
| Colombia | 20.51 (19.19–21.56) | 12.81 (10.76–15.03) | 19.59 (-11.42–50.6) | 17.78 | 16.1 | -1.68 | -1.46 (-1.9–-1.02) | 2.229 (2.228–2.231) |
| Comoros | 43.19 (32.42–56.15) | 43.01 (30.12–55.95) | 54.56 (-124.16–233.29) | 42.16 | 49.98 | 7.82 | -0.03 (-0.36–0.31) | 1.02 (1.018–1.021) |
| Cook Is. | 147.59 (127.53–171.15) | 112.53 (93.14–133.51) | 113.82 (43.02–184.63) | 131.45 | 112.59 | -18.86 | -0.84 (-1.06–-0.61) | 0.034 (0.028–0.04) |
| Costa Rica | 14.49 (13.45–15.4) | 18.56 (16.26–20.93) | 31.56 (-183.8–246.91) | 13.67 | 24 | 10.33 | 1.31 (-1.14–3.82) | 3.184 (2.951–3.418) |
| Côte d'Ivoire | 31.73 (26.61–37.67) | 41.35 (33.01–52.19) | 34.49 (4.19–64.8) | 40.64 | 37.27 | -3.37 | 0.86 (0.65–1.07) | -0.882 (-0.884–-0.88) |
| Croatia | 12.74 (11.7–13.82) | 15.21 (13.3–17.03) | 9.56 (-26.51–45.63) | 13.78 | 11.53 | -2.26 | 0.39 (-0.88–1.68) | -2.058 (-2.069–-2.047) |
| Cuba | 21.41 (20.18–22.43) | 9.71 (8.46–10.97) | 9.82 (-39.06–58.69) | 12.94 | 10.09 | -2.85 | -2.44 (-3.12–-1.75) | -0.16 (-0.164–-0.156) |
| Cyprus | 77.06 (65.9–87.64) | 28.41 (24.15–33.01) | 17.16 (1.76–32.56) | 50.15 | 21.6 | -28.55 | -3.11 (-4–-2.22) | -2.552 (-2.571–-2.534) |
| Czechia | 13.42 (12.19–14.88) | 18.24 (15.67–21.06) | 9.37 (-55.32–74.06) | 11.56 | 13.31 | 1.75 | 1.14 (-1.59–3.94) | -3.513 (-3.553–-3.473) |
| Dem. Rep. Korea | 13.26 (9.99–16.87) | 13.82 (10.77–17.69) | 11.54 (4.64–18.43) | 14.47 | 12.57 | -1.9 | 0.14 (0.11–0.18) | -0.962 (-0.966–-0.958) |
| Democratic Republic of the Congo | 49.76 (39.84–61.06) | 49.74 (37.98–63.7) | 55.77 (27.62–83.92) | 47.78 | 52.8 | 5.02 | -0.02 (-0.18–0.13) | 0.599 (0.585–0.614) |
| Denmark | 9.48 (8.87–10.04) | 9.98 (8.85–10.84) | 6.92 (-15.01–28.85) | 11.29 | 8.43 | -2.86 | 0.2 (-0.71–1.13) | -1.965 (-1.976–-1.954) |
| Djibouti | 35.7 (27.75–47.86) | 46.12 (34.8–62.77) | 47.03 (25.23–68.83) | 42.51 | 46.48 | 3.97 | 0.83 (0.66–1.01) | 0.146 (0.128–0.164) |
| Dominica | 70.92 (64.99–76.68) | 73.09 (63.76–85.11) | 85.44 (33.29–137.59) | 74.22 | 78.47 | 4.25 | 0.08 (-0.11–0.27) | 0.793 (0.771–0.815) |
| Dominican Rep. | 22.69 (19.63–25.56) | 28.04 (22.04–35.1) | 15.34 (-11.95–42.64) | 24.55 | 20.15 | -4.4 | 0.82 (0.22–1.42) | -2.917 (-2.924–-2.911) |
| Ecuador | 22.68 (21.35–23.82) | 28.41 (22.85–34.73) | 18.82 (-40.89–78.53) | 32.19 | 22.74 | -9.45 | 0.56 (-0.77–1.91) | -2.007 (-2.059–-1.956) |
| Egypt | 29.1 (26.08–32.36) | 55.27 (46.65–65.62) | 43.52 (-3.45–90.49) | 43.92 | 50.38 | 6.45 | 2 (1.54–2.45) | -1.394 (-1.416–-1.373) |
| El Salvador | 21.55 (19.49–23.76) | 41.85 (33.3–51.4) | 44.25 (-166.65–255.15) | 33.85 | 43.7 | 9.85 | 2.17 (1.12–3.23) | 0.189 (0.182–0.196) |
| Eq. Guinea | 55.13 (43.47–65.96) | 64.46 (47.28–85.67) | 66.89 (-53.48–187.25) | 55.01 | 65.92 | 10.91 | 0.54 (0.04–1.05) | 0.116 (0.064–0.169) |
| Eritrea | 49.08 (38.23–62.22) | 54.23 (41.63–69.47) | 57.03 (13.45–100.61) | 52.71 | 55.59 | 2.88 | 0.31 (0.14–0.48) | 0.266 (0.243–0.289) |
| Estonia | 3.89 (3.56–4.22) | 10.07 (8.67–11.4) | 7.93 (-51.45–67.31) | 5.68 | 8.66 | 2.98 | 3.11 (1.51–4.74) | -1.285 (-1.339–-1.232) |
| eSwatini | 79.92 (64.61–97.47) | 119.09 (90.49–154.29) | 44.25 (-32.61–121.12) | 124.86 | 71.78 | -53.08 | 1.36 (1.11–1.61) | -4.902 (-4.903–-4.901) |
| Ethiopia | 65.26 (56.84–75.19) | 37 (31.62–43.08) | 42.53 (10.2–74.85) | 47.65 | 40.31 | -7.34 | -1.81 (-1.95–-1.68) | 0.653 (0.651–0.655) |
| Fiji | 192 (163.28–224.45) | 265.22 (213.01–328.29) | 254.72 (112.29–397.15) | 255.13 | 259.72 | 4.58 | 1.02 (0.64–1.4) | -0.253 (-0.325–-0.181) |
| Finland | 6.24 (5.63–6.71) | 3.77 (3.28–4.11) | 3.31 (-3.38–9.99) | 4.4 | 3.62 | -0.78 | -1.67 (-2.48–-0.84) | -0.813 (-0.898–-0.729) |
| France | 8.92 (8.16–9.59) | 6.93 (5.88–7.53) | 4.04 (-5.02–13.11) | 9.14 | 5.39 | -3.74 | -0.72 (-1.25–-0.18) | -2.909 (-2.917–-2.902) |
| Gabon | 63.89 (50.65–81.86) | 74.45 (57.08–95.84) | 62.99 (4.48–121.5) | 72.14 | 68.61 | -3.53 | 0.51 (0.28–0.74) | -0.911 (-1.074–-0.748) |
| Gambia | 26.84 (20.8–33.68) | 43.44 (32.84–55.24) | 44.02 (-101.6–189.63) | 35.96 | 44.09 | 8.13 | 1.6 (1.04–2.16) | -0.002 (-0.009–0.004) |
| Georgia | 10.13 (8.98–11.48) | 16.6 (14.29–18.79) | 10.08 (-58.24–78.39) | 14.99 | 12.75 | -2.24 | 1.76 (-0.03–3.59) | -2.553 (-2.599–-2.506) |
| Germany | 14.97 (13.65–16.03) | 8.99 (7.78–9.75) | 6.28 (-13.83–26.38) | 11.94 | 7.48 | -4.46 | -1.54 (-2.45–-0.62) | -1.928 (-2.041–-1.815) |
| Ghana | 26.88 (22.21–32.87) | 47.27 (37.39–58.08) | 40.06 (-3.46–83.57) | 39.46 | 44.34 | 4.88 | 1.85 (1.71–1.99) | -0.987 (-0.996–-0.979) |
| Greece | 7.8 (7.11–8.37) | 6.19 (5.55–6.7) | 6.55 (-21.55–34.64) | 6.34 | 6.49 | 0.14 | -0.78 (-2.24–0.71) | 0.265 (0.251–0.279) |
| Greenland | 14.92 (12.99–17.06) | 8.93 (7.33–10.98) | 7.11 (1.82–12.4) | 11.66 | 8.13 | -3.54 | -1.56 (-1.76–-1.36) | -1.357 (-1.369–-1.346) |
| Grenada | 83.03 (75.83–90.05) | 81.59 (71.66–90.48) | 73.97 (-159.84–307.79) | 83.82 | 78.56 | -5.26 | 0.2 (-1.24–1.66) | -0.663 (-0.668–-0.657) |
| Guam | 42.98 (38.24–48.08) | 18.73 (16.47–21.16) | 10.42 (-0.15–21) | 27.16 | 13.97 | -13.18 | -2.82 (-3.79–-1.84) | -3.079 (-3.118–-3.04) |
| Guatemala | 19.13 (18.26–19.97) | 62.17 (54.17–70.87) | 41.78 (-201.17–284.73) | 52.28 | 52.55 | 0.27 | 3.69 (1.94–5.48) | -2.303 (-2.322–-2.285) |
| Guinea | 28.31 (20.93–36.51) | 40.35 (31.53–50.81) | 36.19 (16.07–56.31) | 37.26 | 38.25 | 0.99 | 1.17 (1.05–1.29) | -0.607 (-0.612–-0.603) |
| Guinea-Bissau | 41.37 (32.95–50.12) | 53.45 (42.85–63.54) | 47.11 (20.94–73.28) | 50.73 | 50.17 | -0.56 | 0.84 (0.73–0.96) | -0.672 (-0.674–-0.67) |
| Guyana | 77.34 (69.9–84.6) | 83.95 (66.59–104.02) | 68.96 (-53.97–191.9) | 91.7 | 75.92 | -15.78 | 0.34 (-0.43–1.12) | -1.092 (-1.255–-0.928) |
| Haiti | 83.54 (66.47–102.37) | 76.92 (57.14–102.65) | 57.56 (-19.91–135.03) | 78.35 | 66.07 | -12.29 | -0.25 (-0.39–-0.1) | -1.497 (-1.567–-1.426) |
| Honduras | 14.94 (12.55–17.15) | 29.21 (22.89–37.86) | 34.41 (11.1–57.72) | 23.33 | 33.69 | 10.36 | 2.23 (1.58–2.89) | 0.547 (0.508–0.585) |
| Hungary | 12.15 (11.29–13.03) | 12.99 (11.57–14.33) | 6.05 (-23.92–36.01) | 12.64 | 9.01 | -3.64 | 0.48 (-0.92–1.9) | -3.974 (-3.995–-3.954) |
| Iceland | 5.32 (4.83–5.71) | 3.98 (3.36–4.43) | 2.63 (1.04–4.21) | 4.94 | 3.23 | -1.71 | -0.99 (-1.66–-0.31) | -2.229 (-2.237–-2.22) |
| India | 22.01 (18.83–24.87) | 30.31 (26.81–34) | 33.49 (0.81–66.17) | 25.1 | 32.31 | 7.21 | 1.09 (0.43–1.74) | 0.459 (0.453–0.465) |
| Indonesia | 19.67 (16.08–23.07) | 28.62 (23.63–33.11) | 28.34 (15.82–40.87) | 24.99 | 28.88 | 3.89 | 1.22 (1.16–1.27) | -0.105 (-0.117–-0.094) |
| Iran | 13.67 (11.64–16.12) | 20.8 (18.26–22.97) | 11.62 (-8.73–31.98) | 17 | 15.74 | -1.26 | 1.32 (1.17–1.47) | -3.059 (-3.062–-3.057) |
| Iraq | 39.3 (32.28–48.51) | 47.88 (37.91–57.62) | 59.07 (18.4–99.74) | 39.18 | 51.33 | 12.16 | 0.7 (0.2–1.2) | 1.601 (1.601–1.602) |
| Ireland | 10.66 (9.84–11.48) | 4.79 (4.07–5.33) | 1.82 (-0.95–4.6) | 8.1 | 3.04 | -5.06 | -2.55 (-3.38–-1.7) | -4.838 (-4.85–-4.827) |
| Israel | 20.35 (18.79–21.54) | 17.56 (15.01–19.14) | 8.16 (-29.89–46.2) | 26.02 | 12 | -14.02 | -0.52 (-1.52–0.49) | -3.968 (-3.972–-3.964) |
| Italy | 19.45 (17.64–20.4) | 10.25 (8.73–11.11) | 7.69 (-1.64–17.03) | 14.07 | 8.87 | -5.2 | -2.06 (-2.68–-1.44) | -1.524 (-1.524–-1.523) |
| Jamaica | 66.41 (63.14–70.02) | 66.64 (52.28–83.66) | 90.95 (-833.84–1015.74) | 69.98 | 78.67 | 8.7 | 0.14 (-1.62–1.93) | 1.631 (1.621–1.64) |
| Japan | 7.01 (6.43–7.36) | 2.14 (1.85–2.31) | 2.3 (-7.13–11.73) | 3.31 | 2.24 | -1.07 | -3.69 (-4.14–-3.24) | 0.294 (0.259–0.33) |
| Jordan | 53.72 (45.49–63.72) | 37.49 (30.18–45.15) | 47.7 (-41.99–137.38) | 50.68 | 42.71 | -7.96 | -1.11 (-1.83–-0.39) | 1.197 (1.193–1.2) |
| Kazakhstan | 6.42 (5.52–7.45) | 8.45 (7–9.92) | 4.24 (-10.58–19.06) | 9.38 | 5.66 | -3.72 | 0.94 (-0.16–2.05) | -3.029 (-3.041–-3.017) |
| Kenya | 26.99 (21.61–33.43) | 36.75 (30.18–44.62) | 32.65 (8.28–57.01) | 32.75 | 34.58 | 1.82 | 0.99 (0.81–1.18) | -0.579 (-0.581–-0.577) |
| Kiribati | 141.4 (117.4–166.61) | 184.26 (143.26–231.77) | 193.37 (85.01–301.74) | 172.03 | 188.21 | 16.17 | 0.88 (0.82–0.95) | 0.276 (0.273–0.279) |
| Kuwait | 21.41 (18.99–23.15) | 25.1 (20.63–29.95) | 10.85 (-105.7–127.39) | 24.32 | 16.31 | -8.01 | 0.75 (-2.33–3.93) | -4.104 (-4.108–-4.1) |
| Kyrgyzstan | 4.99 (4.51–5.54) | 7.51 (6.28–8.84) | 5.38 (-10.92–21.68) | 7.67 | 6.16 | -1.51 | 1.3 (0.19–2.43) | -1.522 (-1.545–-1.498) |
| Laos | 36.11 (28.32–45.29) | 35.67 (27.93–44.62) | 34.4 (20.69–48.11) | 36.65 | 35.17 | -1.49 | -0.04 (-0.13–0.05) | -0.219 (-0.226–-0.212) |
| Latvia | 5.35 (4.97–5.74) | 13.09 (11.42–14.5) | 7.51 (-38.25–53.27) | 8 | 9.93 | 1.93 | 3.04 (1.51–4.59) | -3.165 (-3.279–-3.05) |
| Lebanon | 36.88 (30.93–44.1) | 29.18 (21.4–36.6) | 31.97 (-3.6–67.54) | 31.8 | 31.13 | -0.67 | -0.73 (-1.11–-0.36) | 0.331 (0.33–0.333) |
| Lesotho | 44.72 (36.75–56.66) | 96.8 (74.38–120.9) | 28.74 (-47.69–105.17) | 79.26 | 52.57 | -26.69 | 2.6 (2.28–2.92) | -5.998 (-6.003–-5.993) |
| Liberia | 30.66 (25.21–36.68) | 42.76 (31.18–57.41) | 50.81 (-2.1–103.72) | 38.5 | 46.77 | 8.27 | 1.07 (0.75–1.39) | 0.878 (0.875–0.882) |
| Libya | 13.18 (10.33–16.12) | 24.22 (17.64–31.87) | 15.86 (-12.69–44.42) | 19.21 | 19.41 | 0.21 | 1.98 (1.56–2.4) | -2.119 (-2.129–-2.109) |
| Lithuania | 3.29 (3.06–3.49) | 9 (7.86–10.17) | 8.64 (-30.77–48.06) | 4.52 | 8.55 | 4.04 | 3.17 (1.76–4.6) | -0.25 (-0.284–-0.216) |
| Luxembourg | 10.95 (10.19–11.57) | 5.91 (5.16–6.56) | 2.79 (-2.51–8.1) | 8.56 | 4.18 | -4.38 | -2.05 (-3.12–-0.97) | -3.942 (-3.951–-3.933) |
| Madagascar | 34.31 (27.81–42.17) | 34.73 (25.91–45.79) | 36.68 (-13.33–86.7) | 33.84 | 35.8 | 1.97 | 0.02 (-0.37–0.42) | 0.267 (0.265–0.269) |
| Malawi | 43.65 (36.33–51.46) | 47.15 (38.79–55.86) | 34.6 (7.03–62.16) | 49.67 | 39.97 | -9.7 | 0.28 (0.14–0.42) | -1.561 (-1.562–-1.559) |
| Malaysia | 26.26 (23.48–29.53) | 19.18 (16.81–21.59) | 30.3 (-31.33–91.93) | 21.81 | 24.08 | 2.27 | -1.07 (-2.2–0.07) | 2.614 (2.587–2.641) |
| Maldives | 33.95 (29.07–39.18) | 17.94 (14.47–21.39) | 12.7 (0.87–24.53) | 24.78 | 14.96 | -9.82 | -2.11 (-2.6–-1.62) | -1.766 (-1.772–-1.76) |
| Mali | 32.47 (27.23–39.21) | 41.52 (33.41–51.4) | 36.14 (17.29–54.99) | 38.59 | 38.98 | 0.39 | 0.82 (0.64–1.01) | -0.792 (-0.793–-0.792) |
| Malta | 24.48 (22.41–26.48) | 13.24 (11.29–14.95) | 25.07 (-102.21–152.35) | 17 | 18.51 | 1.51 | -2.15 (-3.11–-1.17) | 3.496 (3.411–3.581) |
| Marshall Is. | 107.75 (88.63–135.91) | 165.9 (112.7–236.14) | 160.82 (52.33–269.32) | 151.12 | 161.25 | 10.13 | 1.43 (1.34–1.51) | -0.099 (-0.108–-0.091) |
| Mauritania | 29.85 (23.66–36.35) | 36.87 (27.36–49.49) | 54.93 (21.58–88.28) | 32.46 | 46.11 | 13.65 | 0.71 (0.5–0.92) | 2.073 (2.069–2.078) |
| Mauritius | 50.29 (47.64–52.95) | 103.96 (97.02–108.97) | 96.89 (-544.83–738.61) | 85.17 | 96.48 | 11.31 | 2.13 (0.9–3.39) | -0.2 (-0.223–-0.177) |
| Mexico | 71.12 (68.61–73.02) | 67.59 (60.19–75.08) | 82.03 (-190.35–354.41) | 64.55 | 75.42 | 10.88 | 0 (-0.76–0.77) | 0.923 (0.913–0.933) |
| Micronesia | 87.24 (70.44–108.97) | 117.3 (90.97–150.14) | 120.3 (25.78–214.82) | 110.76 | 118.66 | 7.9 | 0.98 (0.92–1.04) | 0.121 (0.117–0.125) |
| Moldova | 6.41 (5.97–6.89) | 8.74 (7.77–9.81) | 7.47 (-39.39–54.32) | 6.62 | 7.59 | 0.96 | 0.96 (-0.85–2.81) | -0.818 (-0.918–-0.717) |
| Monaco | 3.56 (2.76–4.39) | 3.61 (2.89–4.3) | 3.54 (1.56–5.52) | 3.67 | 3.6 | -0.07 | 0.05 (-0.02–0.11) | -0.156 (-0.16–-0.153) |
| Mongolia | 5.41 (4.12–7.05) | 8.53 (6.7–10.67) | 5.17 (-3.62–13.95) | 7.52 | 6.67 | -0.85 | 1.46 (0.74–2.18) | -2.632 (-2.653–-2.61) |
| Montenegro | 11.63 (9.67–13.73) | 17.61 (14.51–20.72) | 13.18 (-8.47–34.82) | 14.49 | 15.4 | 0.9 | 1.43 (1–1.86) | -1.625 (-1.629–-1.621) |
| Morocco | 11.65 (9.7–13.94) | 23.44 (17.43–28.18) | 26.43 (4.54–48.33) | 16.91 | 25.38 | 8.47 | 2.27 (2.05–2.48) | 0.555 (0.539–0.571) |
| Mozambique | 38.2 (33.01–44.96) | 49.25 (37.74–60.72) | 34.68 (7.96–61.41) | 44.98 | 40.95 | -4.03 | 0.86 (0.65–1.07) | -1.838 (-1.843–-1.833) |
| Myanmar | 57.72 (46.32–71.63) | 55.95 (45.71–69.32) | 57.55 (28.23–86.86) | 56.92 | 57.27 | 0.35 | -0.1 (-0.16–-0.04) | 0.11 (0.103–0.118) |
| N. Mariana Is. | 66.33 (55.22–81.83) | 62.07 (54.6–69.18) | 95.11 (-72.42–262.64) | 68.29 | 77.72 | 9.43 | -0.24 (-0.77–0.29) | 2.356 (2.329–2.384) |
| Namibia | 58.99 (49.49–71.52) | 71.87 (55.52–89.78) | 46.22 (-17.94–110.38) | 71.7 | 56.81 | -14.89 | 0.73 (0.43–1.02) | -2.193 (-2.198–-2.188) |
| Nauru | 118.09 (93.73–146.41) | 146.92 (118.07–184.58) | 155.69 (45.04–266.35) | 139.25 | 150.63 | 11.38 | 0.71 (0.64–0.78) | 0.343 (0.341–0.345) |
| Nepal | 20.86 (16.57–25.75) | 31.69 (24.47–40.5) | 36.51 (8.31–64.71) | 24.33 | 34.53 | 10.2 | 1.38 (1.26–1.49) | 0.668 (0.665–0.672) |
| Netherlands | 17.63 (15.85–18.89) | 7.73 (6.58–8.49) | 6.81 (-9.11–22.74) | 12.08 | 7.27 | -4.81 | -2.59 (-3.26–-1.9) | -0.732 (-0.74–-0.725) |
| New Zealand | 10.33 (9.48–11.09) | 7.28 (6.42–7.84) | 6.62 (-17.69–30.94) | 9.9 | 6.67 | -3.24 | -1 (-1.98–-0.02) | -0.326 (-0.351–-0.3) |
| Nicaragua | 24.08 (21.66–26.44) | 29.82 (25.09–35.94) | 17.66 (-3.78–39.09) | 30.61 | 23.67 | -6.94 | 0.52 (-0.63–1.69) | -2.977 (-2.982–-2.972) |
| Niger | 21.36 (16.01–27.36) | 27.13 (20.26–35.96) | 26.05 (7.08–45.03) | 25.22 | 26.92 | 1.7 | 0.8 (0.61–0.98) | -0.304 (-0.307–-0.301) |
| Nigeria | 34.56 (28.8–40.8) | 37.24 (30.81–44.46) | 34.18 (17.27–51.08) | 37 | 35.76 | -1.24 | 0.24 (0.15–0.33) | -0.48 (-0.485–-0.475) |
| Niue | 83.09 (68.68–100.37) | 121.83 (93.46–155.09) | 117.73 (33.22–202.24) | 109.35 | 119.46 | 10.11 | 1.25 (1.09–1.4) | -0.091 (-0.101–-0.081) |
| North Macedonia | 20.41 (16.86–23.89) | 31.61 (25.31–38.26) | 15.77 (0.15–31.38) | 31.81 | 22.31 | -9.5 | 1.51 (1.02–2.01) | -3.52 (-3.521–-3.519) |
| Norway | 6.59 (5.98–6.93) | 5.14 (4.47–5.53) | 3.94 (-4.39–12.27) | 6.93 | 4.52 | -2.4 | -0.97 (-1.9–-0.03) | -1.353 (-1.362–-1.345) |
| Oman | 39.13 (29.83–53.88) | 46.34 (37.05–56.12) | 13.06 (-7.73–33.86) | 46.17 | 24.38 | -21.79 | 0.59 (-0.21–1.39) | -6.177 (-6.19–-6.163) |
| Pakistan | 27.32 (21.95–34.2) | 44.94 (35.73–55.55) | 48.78 (20.69–76.87) | 39.8 | 47.04 | 7.24 | 1.61 (1.43–1.79) | 0.399 (0.398–0.399) |
| Palau | 84.42 (67.73–102.31) | 106.43 (85.51–131.41) | 98.76 (45.71–151.81) | 101.38 | 100.6 | -0.78 | 0.73 (0.54–0.93) | -0.34 (-0.351–-0.328) |
| Palestine | 53.79 (43.14–66.12) | 54.44 (46.57–63.13) | 54.93 (13.25–96.62) | 57.81 | 55.02 | -2.79 | 0 (-0.37–0.37) | -0.018 (-0.124–0.089) |
| Panama | 20.55 (19.08–21.76) | 27.61 (22.02–33.02) | 25.33 (-53.9–104.56) | 26.84 | 27.01 | 0.17 | 1.09 (-0.08–2.26) | -0.711 (-0.742–-0.681) |
| Papua New Guinea | 89.67 (67.63–115.14) | 89.18 (69.47–112.69) | 96.04 (45.81–146.26) | 88.53 | 92.77 | 4.24 | 0 (-0.18–0.18) | 0.349 (0.348–0.35) |
| Paraguay | 25.46 (21.68–29.1) | 56.53 (44.44–70.09) | 33.94 (-38.49–106.37) | 45.69 | 45.3 | -0.39 | 2.71 (1.95–3.48) | -2.892 (-2.998–-2.785) |
| Peru | 14.05 (11.9–16.35) | 17.64 (13.81–22.67) | 9.09 (-25.58–43.75) | 15.39 | 12.76 | -2.63 | 0.83 (-0.45–2.13) | -3.461 (-3.462–-3.459) |
| Philippines | 28.01 (25.57–30.64) | 39.02 (33.52–44.67) | 39.16 (13.05–65.27) | 32.75 | 39.88 | 7.12 | 1.09 (0.69–1.5) | -0.107 (-0.117–-0.097) |
| Poland | 12.39 (11.85–12.86) | 12.91 (11.56–13.98) | 13.02 (-13.31–39.35) | 11.04 | 12.86 | 1.82 | 0.2 (-0.54–0.95) | 0.008 (-0.135–0.151) |
| Portugal | 21.8 (20.23–23.14) | 12.64 (10.88–13.78) | 9.06 (-14.33–32.45) | 19.49 | 10.7 | -8.79 | -1.91 (-2.98–-0.84) | -1.809 (-1.815–-1.804) |
| Puerto Rico | 53.04 (49.82–55.59) | 39.73 (33.08–46.79) | 43.03 (-36.49–122.55) | 50.49 | 42.14 | -8.35 | -0.99 (-1.93–-0.03) | 0.209 (0.202–0.215) |
| Qatar | 85.04 (69.34–104.35) | 76.83 (60.77–95.55) | 44.11 (-27.81–116.03) | 98.74 | 55.47 | -43.28 | -0.29 (-1.44–0.87) | -2.472 (-2.476–-2.468) |
| Republic of Korea | 20.68 (18.66–22.79) | 11.36 (9.41–13) | 8.65 (-2.08–19.39) | 19.69 | 10.04 | -9.65 | -1.95 (-2.38–-1.52) | -1.529 (-1.534–-1.524) |
| Republic of the Congo | 65.7 (54.02–78.54) | 63.51 (51.8–78.48) | 57.52 (-44.24–159.27) | 65.68 | 60.28 | -5.39 | -0.09 (-0.29–0.11) | -0.53 (-0.54–-0.521) |
| Romania | 7.01 (6.56–7.55) | 6.8 (6.06–7.64) | 6.71 (-7.16–20.58) | 6.22 | 6.87 | 0.64 | -0.13 (-0.97–0.72) | -0.069 (-0.086–-0.051) |
| Russia | 3.71 (3.58–3.8) | 15.64 (14.41–16.85) | 10.03 (-54.04–74.1) | 6.59 | 12.2 | 5.61 | 4.83 (3.17–6.53) | -2.027 (-2.03–-2.023) |
| Rwanda | 56.91 (41.34–75.32) | 41.63 (26.63–58.53) | 46.26 (-16.42–108.93) | 49.48 | 44.37 | -5.1 | -1.01 (-1.14–-0.87) | 0.546 (0.54–0.553) |
| S. Sudan | 45.78 (33.3–61.06) | 56.22 (41.94–75.96) | 65.77 (-12.39–143.93) | 48.66 | 60.95 | 12.29 | 0.67 (0.55–0.79) | 0.774 (0.773–0.776) |
| Saint Kitts and Nevis | 72.93 (68.03–77.75) | 51.64 (43.38–59.14) | 52.21 (-131.23–235.65) | 58.84 | 54.33 | -4.51 | -1.04 (-1.81–-0.28) | -0.237 (-0.257–-0.217) |
| Saint Lucia | 97.43 (92.37–102.71) | 57.11 (47.82–67.21) | 91.3 (-94.81–277.41) | 74.41 | 72.21 | -2.2 | -1.91 (-2.66–-1.16) | 2.664 (2.624–2.704) |
| Samoa | 75.78 (63.81–90.78) | 92.4 (74.37–114.94) | 96.58 (37.92–155.24) | 85.86 | 94 | 8.14 | 0.65 (0.56–0.73) | 0.2 (0.189–0.21) |
| San Marino | 7.8 (6.58–9.09) | 3.74 (2.53–5.12) | 0.14 (-0.62–0.91) | 6.49 | 1.02 | -5.48 | -2.62 (-2.83–-2.42) | -15.686 (-15.687–-15.684) |
| Sao Tome and Principe | 12.54 (10.91–14.44) | 17.91 (15.26–20.98) | 18.3 (7.32–29.27) | 15.97 | 18.32 | 2.35 | 1.16 (0.89–1.44) | 0.036 (0.03–0.041) |
| Saudi Arabia | 23.23 (17.36–30.82) | 29.33 (23.27–35.25) | 33.05 (-10.32–76.42) | 28.12 | 31.08 | 2.96 | 0.71 (0.53–0.89) | 0.534 (0.525–0.542) |
| Senegal | 30.79 (25.59–36.86) | 45.64 (35.58–58.05) | 39.44 (-117–195.88) | 39.72 | 42.01 | 2.29 | 1.29 (0.95–1.64) | -0.659 (-0.662–-0.656) |
| Serbia | 20.87 (18.14–24.15) | 21.16 (17.78–25.32) | 18.8 (-8.62–46.21) | 22.16 | 20.09 | -2.07 | 0.07 (-0.74–0.89) | -0.677 (-0.712–-0.642) |
| Seychelles | 16.16 (14.38–17.93) | 24.13 (21.06–27.75) | 10.13 (-7.23–27.49) | 22.07 | 15.59 | -6.48 | 1.22 (0.43–2.03) | -4.426 (-4.487–-4.365) |
| Sierra Leone | 23.84 (19.25–29.07) | 32.45 (25.56–41.85) | 30.46 (11.79–49.13) | 30.45 | 31.34 | 0.89 | 1 (0.86–1.15) | -0.344 (-0.348–-0.339) |
| Singapore | 17.62 (16.42–18.76) | 1.98 (1.73–2.17) | 0.96 (-2.94–4.87) | 9.05 | 1.32 | -7.73 | -6.72 (-8.56–-4.83) | -3.55 (-3.603–-3.497) |
| Slovakia | 11.26 (9.84–12.94) | 8.44 (6.8–10.1) | 7.4 (2.43–12.37) | 9.58 | 7.88 | -1.7 | -0.92 (-1.43–-0.42) | -0.666 (-0.667–-0.664) |
| Slovenia | 10.52 (9.69–11.44) | 8.21 (7.09–9.49) | 4 (-17.44–25.43) | 11.88 | 5.54 | -6.34 | -0.72 (-2.64–1.23) | -3.516 (-3.529–-3.503) |
| Solomon Is. | 84.29 (60.58–113.29) | 111.6 (89.17–139.1) | 102.87 (1.28–204.47) | 96.63 | 106.48 | 9.85 | 0.91 (0.41–1.42) | -0.444 (-0.448–-0.439) |
| Somalia | 51.33 (39.97–63.89) | 53.7 (40.62–65.51) | 39.55 (18.33–60.77) | 55.12 | 46.04 | -9.08 | 0.16 (0.09–0.22) | -1.639 (-1.65–-1.629) |
| South Africa | 42.66 (38.42–46.65) | 77.34 (71.8–82.79) | 74.15 (-30.14–178.45) | 71.7 | 79.15 | 7.45 | 1.95 (1.2–2.69) | -0.562 (-0.581–-0.544) |
| Spain | 17.66 (15.73–18.83) | 6.96 (5.82–7.68) | 4.24 (-0.77–9.25) | 11.67 | 5.47 | -6.19 | -2.88 (-3.38–-2.37) | -2.692 (-2.702–-2.682) |
| Sri Lanka | 38.03 (32.84–42.67) | 47.03 (33.11–62.12) | 56.84 (-92.95–206.62) | 45.28 | 54.28 | 9 | 0.68 (0.06–1.3) | 0.608 (0.6–0.615) |
| St. Vin. and Gren. | 98.01 (90.45–104.18) | 69.95 (62.41–78.14) | 65.96 (-166.04–297.95) | 87.08 | 67.9 | -19.18 | -1.01 (-2.19–0.19) | -0.37 (-0.374–-0.367) |
| Sudan | 11.98 (9.61–15.9) | 17.94 (13.76–23.37) | 24.27 (7.75–40.78) | 14.14 | 21.2 | 7.06 | 1.32 (1.24–1.39) | 1.581 (1.58–1.583) |
| Suriname | 39.32 (35.64–43.01) | 41.74 (32.59–52.05) | 11 (-29.04–51.04) | 42.17 | 22.66 | -19.51 | 0.32 (-0.32–0.96) | -6.936 (-6.947–-6.925) |
| Sweden | 8.73 (7.83–9.33) | 7.25 (6.13–8.24) | 4.12 (-4.41–12.65) | 8.54 | 5.66 | -2.88 | -0.67 (-1.48–0.15) | -3.255 (-3.257–-3.253) |
| Switzerland | 13.71 (12.24–14.7) | 5.19 (4.36–5.71) | 3.58 (-0.74–7.89) | 9.54 | 4.36 | -5.19 | -3.13 (-3.97–-2.29) | -2.081 (-2.149–-2.012) |
| Syria | 18.14 (14.8–21.87) | 19.76 (15–25.73) | 19.67 (-9.69–49.03) | 19.83 | 20.33 | 0.5 | 0.3 (0–0.59) | -0.267 (-0.272–-0.261) |
| Tajikistan | 11.77 (10.08–13.88) | 13.58 (10.32–17.32) | 9.56 (-15.24–34.35) | 15.36 | 11.69 | -3.67 | 0.43 (-0.02–0.89) | -2.058 (-2.058–-2.058) |
| Tanzania | 36.77 (31.31–42.69) | 37.46 (30.1–47.33) | 39.44 (17.66–61.23) | 38.22 | 38.56 | 0.35 | 0.06 (-0.05–0.18) | 0.265 (0.264–0.265) |
| Thailand | 23.59 (19.3–28.62) | 22.28 (16.87–27.89) | 29.74 (-5.49–64.98) | 23.89 | 25.91 | 2.02 | -0.16 (-0.5–0.17) | 1.518 (1.516–1.519) |
| Timor-Leste | 18.54 (13.75–23.92) | 22.12 (16.96–28.97) | 23.69 (6.56–40.83) | 19.15 | 23.15 | 4 | 0.54 (0.29–0.79) | 0.272 (0.264–0.28) |
| Togo | 24.16 (19.54–29.76) | 35.17 (27.24–45.65) | 33.64 (7.83–59.44) | 31.97 | 34.41 | 2.44 | 1.25 (1–1.5) | -0.224 (-0.234–-0.213) |
| Tonga | 90.89 (76.12–105.87) | 111.25 (89.65–138.14) | 115.83 (-19.2–250.87) | 104.53 | 113.23 | 8.71 | 0.67 (0.5–0.84) | 0.182 (0.169–0.196) |
| Trinidad and Tobago | 129.20 (123.48–135.38) | 95.70 (74.35–118.72) | 84.32 (-97.63–266.27) | 110.3 | 87.05 | -23.25 | -1 (-1.9–-0.1) | -0.494 (-0.499–-0.488) |
| Tunisia | 10.36 (8.74–12.21) | 16.51 (12.09–22.19) | 18.38 (5.89–30.86) | 13.78 | 17.49 | 3.71 | 1.5 (1.2–1.81) | 0.516 (0.506–0.526) |
| Turkey | 35.78 (30.65–42.17) | 26.32 (21.29–32.01) | 22.03 (-17.96–62.02) | 27.96 | 24.49 | -3.47 | -0.97 (-1.67–-0.25) | -1.10 (-1.107–-1.092) |
| Turkmenistan | 9.54 (8.45–10.72) | 20.47 (15.82–26.5) | 19.68 (-72.32–111.67) | 16.23 | 19.35 | 3.12 | 2.67 (1.15–4.21) | -0.177 (-0.202–-0.151) |
| Tuvalu | 86.89 (71.25–108.62) | 100.19 (81.47–124.02) | 95.22 (44.28–146.15) | 97.5 | 97.46 | -0.04 | 0.47 (0.41–0.53) | -0.297 (-0.303–-0.291) |
| U.S. Virgin Is. | 43.68 (37.45–51.05) | 28.29 (22.69–34.77) | 11.41 (-5.37–28.19) | 39.19 | 17.72 | -21.47 | -1.46 (-1.83–-1.09) | -4.663 (-4.676–-4.65) |
| Uganda | 40.94 (28.62–63.75) | 46.13 (32.97–64.53) | 47.94 (10.59–85.29) | 47.36 | 46.96 | -0.4 | 0.37 (0.22–0.51) | 0.2 (0.193–0.207) |
| Ukraine | 3.34 (3.09–3.56) | 2.88 (2.13–3.67) | 2.21 (-7.98–12.39) | 3.37 | 2.63 | -0.73 | -0.57 (-2.31–1.2) | -1.742 (-1.754–-1.73) |
| United Arab Emirates | 41.78 (32.15–55.32) | 42.78 (33.24–51.86) | 7.75 (-8.69–24.19) | 53.3 | 20.41 | -32.89 | 0.07 (-2.12–2.3) | -8.823 (-8.833–-8.813) |
| United Kingdom | 8.7 (8.12–9) | 4.13 (3.66–4.36) | 2.75 (-4.56–10.05) | 5.82 | 3.37 | -2.44 | -2.52 (-3.59–-1.44) | -1.95 (-2.015–-1.885) |
| United States | 14.29 (13.19–14.94) | 11.81 (10.83–12.49) | 13.59 (-4.42–31.61) | 14.99 | 12.67 | -2.31 | -0.62 (-0.89–-0.35) | 0.746 (0.741–0.752) |
| Uruguay | 16.39 (15.31–17.42) | 15.69 (14.16–16.84) | 16.41 (-0.74–33.56) | 15.94 | 16.2 | 0.27 | -0.17 (-0.95–0.61) | 0.199 (0.173–0.226) |
| Uzbekistan | 9.07 (8.45–9.82) | 24.03 (20.26–28.1) | 15.93 (-46.81–78.68) | 20.41 | 19.29 | -1.11 | 3.16 (2–4.33) | -2.071 (-2.132–-2.009) |
| Vanuatu | 71.49 (55.19–102.06) | 89.38 (73.03–111.59) | 98.85 (43.03–154.66) | 82.2 | 94.21 | 12.01 | 0.73 (0.52–0.94) | 0.498 (0.494–0.503) |
| Venezuela | 33.77 (31.59–35.53) | 43.01 (33.52–54.69) | 53.78 (-103.81–211.36) | 34.95 | 47.69 | 12.74 | 1.08 (-0.02–2.18) | 1.29 (1.286–1.294) |
| Vietnam | 29.8 (24.19–36.82) | 35.56 (28.97–42.68) | 34.99 (18.32–51.66) | 32.39 | 35.52 | 3.13 | 0.59 (0.53–0.65) | -0.164 (-0.164–-0.164) |
| Yemen | 11.79 (8.91–16.9) | 14.45 (10.24–21.51) | 18.97 (5–32.95) | 12.57 | 16.7 | 4.13 | 0.67 (0.52–0.81) | 1.472 (1.472–1.473) |
| Zambia | 47.58 (40.2–57.25) | 46.13 (35.74–57.73) | 28.42 (1.85–54.99) | 51.6 | 35.9 | -15.7 | -0.06 (-0.26–0.15) | -2.474 (-2.476–-2.471) |
| Zimbabwe | 37.08 (30.55–43.9) | 65.75 (51.75–81.44) | 21.45 (-39.64–82.53) | 58.93 | 36.89 | -22.04 | 2 (1.6–2.39) | -5.423 (-5.425–-5.421) |

- 1. Supplementary Table S7. The frontier analysis based on socio-demographic index and age-standardized mortality rate for T2DM in 2021 and 2040 in 204 countries and territories to obtain the frontier line (optimal age-standardized mortality rate corresponding to each socio-demographic index).

| **Country** （Territory） | **Socio-Demographic Index** | | **ASMR** | | **Frontier line** | |
| --- | --- | --- | --- | --- | --- | --- |
|  | **2021** | **2040** | **2021** | **2040** | **2021** | **2040** |
| Afghanistan | 0.34 | 0.48 | 39.34 | 47.37 | 30.1 | 23.93 |
| Albania | 0.71 | 0.76 | 4.67 | 4.09 | 4.67 | 4.09 |
| Algeria | 0.66 | 0.78 | 18.54 | 24.5 | 8.41 | 4.67 |
| American Samoa | 0.72 | 0.77 | 112.78 | 78.93 | 5.84 | 4.64 |
| Andorra | 0.87 | 0.92 | 8.29 | 2.66 | 2.98 | 1.23 |
| Angola | 0.45 | 0.57 | 48.2 | 53.29 | 18.62 | 20.39 |
| Antigua and Barb. | 0.75 | 0.82 | 57.21 | 23.21 | 5.81 | 3.03 |
| Argentina | 0.72 | 0.81 | 15.97 | 6.23 | 5.69 | 4.28 |
| Armenia | 0.7 | 0.78 | 14.12 | 11.08 | 8.14 | 4.65 |
| Australia | 0.84 | 0.91 | 8.42 | 8.64 | 3.56 | 1.3 |
| Austria | 0.85 | 0.91 | 9.09 | 3.25 | 3.5 | 1.62 |
| Azerbaijan | 0.69 | 0.74 | 16.94 | 7.84 | 8.17 | 5.55 |
| Bahamas | 0.81 | 0.88 | 35.68 | 30.46 | 3.49 | 2.9 |
| Bahrain | 0.75 | 0.86 | 126.21 | 99.88 | 5.82 | 2.93 |
| Bangladesh | 0.49 | 0.68 | 35.06 | 35.58 | 18.42 | 11.2 |
| Barbados | 0.75 | 0.79 | 59.37 | 30.75 | 5.84 | 4.36 |
| Belarus | 0.78 | 0.88 | 4.01 | 4.56 | 3.31 | 2.91 |
| Belgium | 0.85 | 0.91 | 5.01 | 2.72 | 3.47 | 1.23 |
| Belize | 0.61 | 0.68 | 53.99 | 34.62 | 9.86 | 11.32 |
| Benin | 0.37 | 0.51 | 36 | 37.75 | 29.36 | 22.69 |
| Bermuda | 0.82 | 0.86 | 16.58 | 15.8 | 3.58 | 2.99 |
| Bhutan | 0.47 | 0.56 | 31.34 | 32.44 | 18.31 | 20.33 |
| Bolivia | 0.6 | 0.7 | 42.27 | 39.4 | 14.02 | 5.92 |
| Bosnia and Herz. | 0.72 | 0.8 | 30.06 | 10.5 | 5.8 | 4.33 |
| Botswana | 0.64 | 0.71 | 59.85 | 33.17 | 8.46 | 5.77 |
| Brazil | 0.65 | 0.73 | 25.83 | 21.21 | 8.57 | 5.76 |
| Brunei | 0.81 | 0.86 | 49.92 | 26.26 | 3.56 | 2.98 |
| Bulgaria | 0.77 | 0.86 | 15.59 | 12.31 | 4.04 | 2.96 |
| Burkina Faso | 0.29 | 0.4 | 33.41 | 27.33 | 29.46 | 26.53 |
| Burundi | 0.29 | 0.34 | 44.17 | 45.94 | 30.24 | 30.89 |
| Côte d'Ivoire | 0.43 | 0.52 | 41.35 | 34.49 | 29.45 | 20.4 |
| Cabo Verde | 0.53 | 0.66 | 35.47 | 29.92 | 16.81 | 11.19 |
| Cambodia | 0.47 | 0.59 | 32.01 | 36.13 | 18.06 | 20.23 |
| Cameroon | 0.48 | 0.64 | 50.33 | 44.5 | 18.54 | 11.09 |
| Canada | 0.87 | 0.92 | 7.53 | 5.04 | 2.58 | 1.24 |
| Central African Rep. | 0.31 | 0.37 | 61.79 | 52.53 | 30.31 | 30.93 |
| Chad | 0.24 | 0.32 | 35.47 | 36.45 | 30.2 | 29.88 |
| Chile | 0.77 | 0.87 | 10.91 | 7.94 | 3.91 | 2.94 |
| China | 0.72 | 0.88 | 8.74 | 7.2 | 5.78 | 2.91 |
| Colombia | 0.66 | 0.73 | 12.81 | 19.59 | 8.32 | 5.7 |
| Comoros | 0.48 | 0.55 | 43.01 | 54.56 | 18.32 | 20.32 |
| Cook Is. | 0.78 | 0.89 | 112.53 | 113.82 | 3.87 | 2.88 |
| Costa Rica | 0.7 | 0.83 | 18.56 | 31.56 | 8.22 | 3.01 |
| Croatia | 0.8 | 0.87 | 15.21 | 9.56 | 3.53 | 2.97 |
| Cuba | 0.67 | 0.74 | 9.71 | 9.82 | 8.07 | 5.8 |
| Cyprus | 0.84 | 0.9 | 28.41 | 17.16 | 3.6 | 1.95 |
| Czechia | 0.83 | 0.88 | 18.24 | 9.37 | 3.51 | 2.95 |
| Dem. Rep. Korea | 0.57 | 0.61 | 13.82 | 11.54 | 13.66 | 11.54 |
| Democratic Republic of the Congo | 0.38 | 0.47 | 49.74 | 55.77 | 29.39 | 28 |
| Denmark | 0.9 | 0.96 | 9.98 | 6.92 | 2.58 | 1.21 |
| Djibouti | 0.49 | 0.62 | 46.12 | 47.03 | 18.58 | 14.09 |
| Dominica | 0.75 | 0.81 | 73.09 | 85.44 | 5.82 | 4.31 |
| Dominican Rep. | 0.62 | 0.71 | 28.04 | 15.34 | 8.48 | 5.83 |
| Ecuador | 0.66 | 0.75 | 28.41 | 18.82 | 8.47 | 5.64 |
| Egypt | 0.61 | 0.73 | 55.27 | 43.52 | 9.85 | 5.74 |
| El Salvador | 0.56 | 0.68 | 41.85 | 44.25 | 14.49 | 11.2 |
| Eq. Guinea | 0.66 | 0.77 | 64.46 | 66.89 | 8.43 | 4.61 |
| Eritrea | 0.4 | 0.49 | 54.23 | 57.03 | 29.27 | 22.71 |
| Estonia | 0.84 | 0.9 | 10.07 | 7.93 | 3.59 | 1.72 |
| eSwatini | 0.59 | 0.63 | 119.09 | 44.25 | 13.99 | 11.06 |
| Ethiopia | 0.36 | 0.44 | 37 | 42.53 | 29.57 | 28.2 |
| Fiji | 0.68 | 0.75 | 265.22 | 254.72 | 8.15 | 5.62 |
| Finland | 0.86 | 0.92 | 3.77 | 3.31 | 2.91 | 1.24 |
| France | 0.84 | 0.89 | 6.93 | 4.04 | 3.5 | 1.92 |
| Gabon | 0.63 | 0.78 | 74.45 | 62.99 | 8.49 | 4.68 |
| Gambia | 0.41 | 0.52 | 43.44 | 44.02 | 29.53 | 22.83 |
| Georgia | 0.73 | 0.81 | 16.6 | 10.08 | 5.79 | 4.3 |
| Germany | 0.9 | 0.95 | 8.99 | 6.28 | 2.55 | 1.21 |
| Ghana | 0.56 | 0.71 | 47.27 | 40.06 | 14.5 | 5.84 |
| Greece | 0.79 | 0.84 | 6.19 | 6.55 | 3.58 | 3.05 |
| Greenland | 0.83 | 0.84 | 8.93 | 7.11 | 3.58 | 3.02 |
| Grenada | 0.67 | 0.77 | 81.59 | 73.97 | 8.15 | 4.65 |
| Guam | 0.8 | 0.87 | 18.73 | 10.42 | 3.55 | 2.99 |
| Guatemala | 0.54 | 0.67 | 62.17 | 41.78 | 16.57 | 11.18 |
| Guinea | 0.34 | 0.48 | 40.35 | 36.19 | 29.95 | 23.68 |
| Guinea-Bissau | 0.35 | 0.47 | 53.45 | 47.11 | 30.09 | 27.86 |
| Guyana | 0.65 | 0.77 | 83.95 | 68.96 | 8.63 | 4.59 |
| Haiti | 0.45 | 0.48 | 76.92 | 57.56 | 24.9 | 23.69 |
| Honduras | 0.51 | 0.6 | 29.21 | 34.41 | 16.66 | 18.83 |
| Hungary | 0.79 | 0.87 | 12.99 | 6.05 | 3.55 | 2.96 |
| Iceland | 0.88 | 0.95 | 3.98 | 2.63 | 2.58 | 1.2 |
| India | 0.58 | 0.71 | 30.31 | 33.49 | 13.95 | 5.77 |
| Indonesia | 0.66 | 0.78 | 28.62 | 28.34 | 8.4 | 4.56 |
| Iran | 0.7 | 0.81 | 20.8 | 11.62 | 8.15 | 4.29 |
| Iraq | 0.66 | 0.84 | 47.88 | 59.07 | 8.43 | 2.92 |
| Ireland | 0.87 | 0.9 | 4.79 | 1.82 | 2.6 | 1.38 |
| Israel | 0.81 | 0.88 | 17.56 | 8.16 | 3.53 | 2.96 |
| Italy | 0.81 | 0.86 | 10.25 | 7.69 | 3.51 | 2.94 |
| Jamaica | 0.68 | 0.75 | 66.64 | 90.95 | 8.12 | 5.58 |
| Japan | 0.87 | 0.92 | 2.14 | 2.3 | 2.1 | 1.23 |
| Jordan | 0.73 | 0.83 | 37.49 | 47.7 | 5.9 | 2.97 |
| Kazakhstan | 0.73 | 0.79 | 8.45 | 4.24 | 5.74 | 4.14 |
| Kenya | 0.52 | 0.69 | 36.75 | 32.65 | 16.7 | 7.38 |
| Kiribati | 0.53 | 0.6 | 184.26 | 193.37 | 16.62 | 18.84 |
| Kuwait | 0.85 | 0.89 | 25.1 | 10.85 | 3.52 | 2.86 |
| Kyrgyzstan | 0.6 | 0.7 | 7.51 | 5.38 | 7.51 | 5.25 |
| Laos | 0.49 | 0.58 | 35.67 | 34.4 | 18.48 | 20.43 |
| Latvia | 0.83 | 0.92 | 13.09 | 7.51 | 3.54 | 1.21 |
| Lebanon | 0.74 | 0.78 | 29.18 | 31.97 | 5.73 | 4.6 |
| Lesotho | 0.51 | 0.61 | 96.8 | 28.74 | 16.64 | 18.71 |
| Liberia | 0.35 | 0.43 | 42.76 | 50.81 | 29.92 | 27.77 |
| Libya | 0.73 | 0.83 | 24.22 | 15.86 | 5.8 | 2.91 |
| Lithuania | 0.86 | 0.91 | 9 | 8.64 | 3.09 | 1.71 |
| Luxembourg | 0.88 | 0.92 | 5.91 | 2.79 | 2.54 | 1.23 |
| Madagascar | 0.4 | 0.56 | 34.73 | 36.68 | 29.31 | 20.31 |
| Malawi | 0.38 | 0.54 | 47.15 | 34.6 | 29.38 | 20.41 |
| Malaysia | 0.74 | 0.84 | 19.18 | 30.3 | 5.75 | 2.94 |
| Maldives | 0.65 | 0.7 | 17.94 | 12.7 | 8.49 | 5.79 |
| Mali | 0.27 | 0.39 | 41.52 | 36.14 | 31.3 | 29.88 |
| Malta | 0.8 | 0.87 | 13.24 | 25.07 | 3.5 | 2.92 |
| Marshall Is. | 0.57 | 0.67 | 165.9 | 160.82 | 14.01 | 11.07 |
| Mauritania | 0.5 | 0.64 | 36.87 | 54.93 | 18.55 | 11.17 |
| Mauritius | 0.72 | 0.82 | 103.96 | 96.89 | 5.87 | 2.98 |
| Mexico | 0.66 | 0.76 | 67.59 | 82.03 | 8.49 | 4.58 |
| Micronesia | 0.59 | 0.65 | 117.3 | 120.3 | 14 | 11.02 |
| Moldova | 0.73 | 0.84 | 8.74 | 7.47 | 5.73 | 2.91 |
| Monaco | 0.91 | 0.94 | 3.61 | 3.54 | 2.5 | 1.26 |
| Mongolia | 0.62 | 0.69 | 8.53 | 5.17 | 7.88 | 5.17 |
| Montenegro | 0.8 | 0.85 | 17.61 | 13.18 | 3.53 | 2.98 |
| Morocco | 0.56 | 0.71 | 23.44 | 26.43 | 14.58 | 5.86 |
| Mozambique | 0.33 | 0.48 | 49.25 | 34.68 | 30.18 | 23.87 |
| Myanmar | 0.53 | 0.57 | 55.95 | 57.55 | 16.5 | 20.34 |
| N. Mariana Is. | 0.77 | 0.86 | 62.07 | 95.11 | 3.85 | 2.93 |
| Namibia | 0.62 | 0.71 | 71.87 | 46.22 | 9.87 | 5.82 |
| Nauru | 0.63 | 0.74 | 146.92 | 155.69 | 8.67 | 5.83 |
| Nepal | 0.43 | 0.55 | 31.69 | 36.51 | 28.93 | 20.38 |
| Netherlands | 0.89 | 0.94 | 7.73 | 6.81 | 2.57 | 1.21 |
| New Zealand | 0.85 | 0.91 | 7.28 | 6.62 | 3.52 | 1.7 |
| Nicaragua | 0.52 | 0.59 | 29.82 | 17.66 | 16.7 | 17.66 |
| Niger | 0.17 | 0.24 | 27.13 | 26.05 | 27.13 | 26.05 |
| Nigeria | 0.5 | 0.64 | 37.24 | 34.18 | 18.55 | 11.2 |
| Niue | 0.73 | 0.8 | 121.83 | 117.73 | 5.76 | 4.32 |
| North Macedonia | 0.75 | 0.83 | 31.61 | 15.77 | 5.84 | 2.97 |
| Norway | 0.92 | 0.94 | 5.14 | 3.94 | 2.52 | 1.15 |
| Oman | 0.77 | 0.87 | 46.34 | 13.06 | 3.86 | 2.94 |
| Pakistan | 0.5 | 0.62 | 44.94 | 48.78 | 18.48 | 14.11 |
| Palau | 0.75 | 0.77 | 106.43 | 98.76 | 5.87 | 4.67 |
| Palestine | 0.63 | 0.77 | 54.44 | 54.93 | 8.55 | 4.59 |
| Panama | 0.71 | 0.88 | 27.61 | 25.33 | 5.8 | 2.94 |
| Papua New Guinea | 0.42 | 0.48 | 89.18 | 96.04 | 29.3 | 23.66 |
| Paraguay | 0.64 | 0.73 | 56.53 | 33.94 | 8.46 | 5.68 |
| Peru | 0.66 | 0.73 | 17.64 | 9.09 | 8.52 | 5.74 |
| Philippines | 0.65 | 0.8 | 39.02 | 39.16 | 8.47 | 4.32 |
| Poland | 0.81 | 0.89 | 12.91 | 13.02 | 3.54 | 1.95 |
| Portugal | 0.74 | 0.8 | 12.64 | 9.06 | 5.73 | 4.31 |
| Puerto Rico | 0.83 | 0.92 | 39.73 | 43.03 | 3.51 | 1.18 |
| Qatar | 0.85 | 0.94 | 76.83 | 44.11 | 3.47 | 1.23 |
| Republic of Korea | 0.89 | 0.95 | 11.36 | 8.65 | 2.55 | 1.22 |
| Republic of the Congo | 0.58 | 0.67 | 63.51 | 57.52 | 14.01 | 11.13 |
| Romania | 0.77 | 0.86 | 6.8 | 6.71 | 3.9 | 2.95 |
| Russia | 0.81 | 0.89 | 15.64 | 10.03 | 3.53 | 1.99 |
| Rwanda | 0.44 | 0.59 | 41.63 | 46.26 | 29.24 | 20.41 |
| S. Sudan | 0.28 | 0.31 | 56.22 | 65.77 | 31.92 | 35.07 |
| Saint Kitts and Nevis | 0.75 | 0.83 | 51.64 | 52.21 | 5.8 | 2.94 |
| Saint Lucia | 0.67 | 0.74 | 57.11 | 91.3 | 8.09 | 5.6 |
| Samoa | 0.59 | 0.66 | 92.4 | 96.58 | 14.07 | 11.32 |
| San Marino | 0.89 | 0.91 | 3.74 | 0.14 | 2.53 | 0.14 |
| Sao Tome and Principe | 0.51 | 0.61 | 17.91 | 18.3 | 15.78 | 17.9 |
| Saudi Arabia | 0.82 | 0.9 | 29.33 | 33.05 | 3.51 | 1.73 |
| Senegal | 0.41 | 0.55 | 45.64 | 39.44 | 29.5 | 20.34 |
| Serbia | 0.79 | 0.87 | 21.16 | 18.8 | 3.5 | 2.95 |
| Seychelles | 0.73 | 0.83 | 24.13 | 10.13 | 5.7 | 2.95 |
| Sierra Leone | 0.36 | 0.49 | 32.45 | 30.46 | 29.25 | 22.64 |
| Singapore | 0.86 | 0.89 | 1.98 | 0.96 | 1.98 | 0.96 |
| Slovakia | 0.81 | 0.86 | 8.44 | 7.4 | 3.53 | 2.93 |
| Slovenia | 0.84 | 0.88 | 8.21 | 4 | 3.49 | 2.8 |
| Solomon Is. | 0.43 | 0.5 | 111.6 | 102.87 | 29.37 | 22.74 |
| Somalia | 0.08 | 0.1 | 53.7 | 39.55 | 53.7 | 39.55 |
| South Africa | 0.68 | 0.72 | 77.34 | 74.15 | 8.08 | 5.8 |
| Spain | 0.77 | 0.82 | 6.96 | 4.24 | 3.95 | 2.96 |
| Sri Lanka | 0.7 | 0.81 | 47.03 | 56.84 | 8.14 | 4.29 |
| St. Vin. and Gren. | 0.64 | 0.74 | 69.95 | 65.96 | 8.56 | 5.73 |
| Sudan | 0.54 | 0.7 | 17.94 | 24.27 | 14.4 | 5.89 |
| Suriname | 0.63 | 0.69 | 41.74 | 11 | 8.5 | 6.89 |
| Sweden | 0.89 | 0.94 | 7.25 | 4.12 | 2.56 | 1.2 |
| Switzerland | 0.93 | 0.97 | 5.19 | 3.58 | 2.53 | 1.28 |
| Syria | 0.62 | 0.74 | 19.76 | 19.67 | 8.56 | 5.62 |
| Tajikistan | 0.54 | 0.63 | 13.58 | 9.56 | 13.58 | 9.56 |
| Tanzania | 0.45 | 0.61 | 37.46 | 39.44 | 24.88 | 14.08 |
| Thailand | 0.68 | 0.75 | 22.28 | 29.74 | 8.16 | 5.62 |
| Timor-Leste | 0.44 | 0.49 | 22.12 | 23.69 | 22.12 | 22.27 |
| Togo | 0.41 | 0.55 | 35.17 | 33.64 | 29.22 | 20.52 |
| Tonga | 0.63 | 0.73 | 111.25 | 115.83 | 8.6 | 5.89 |
| Trinidad and Tobago | 0.77 | 0.81 | 95.7 | 84.32 | 3.98 | 4.29 |
| Tunisia | 0.68 | 0.77 | 16.51 | 18.38 | 8.09 | 4.57 |
| Turkey | 0.71 | 0.84 | 26.32 | 22.03 | 5.83 | 2.96 |
| Turkmenistan | 0.68 | 0.75 | 20.47 | 19.68 | 8.14 | 5.58 |
| Tuvalu | 0.58 | 0.67 | 100.19 | 95.22 | 14.08 | 11.35 |
| U.S. Virgin Is. | 0.82 | 0.87 | 28.29 | 11.41 | 3.55 | 2.98 |
| Uganda | 0.42 | 0.59 | 46.13 | 47.94 | 29.27 | 20.44 |
| Ukraine | 0.76 | 0.82 | 2.88 | 2.21 | 2.88 | 2.21 |
| United Arab Emirates | 0.85 | 0.91 | 42.78 | 7.75 | 3.51 | 1.64 |
| United Kingdom | 0.86 | 0.93 | 4.13 | 2.75 | 3.01 | 1.2 |
| United States | 0.86 | 0.92 | 11.81 | 13.59 | 2.99 | 1.23 |
| Uruguay | 0.72 | 0.8 | 15.69 | 16.41 | 5.85 | 4.31 |
| Uzbekistan | 0.66 | 0.76 | 24.03 | 15.93 | 8.54 | 4.61 |
| Vanuatu | 0.47 | 0.55 | 89.38 | 98.85 | 18.45 | 20.48 |
| Venezuela | 0.6 | 0.58 | 43.01 | 53.78 | 14.05 | 20.31 |
| Vietnam | 0.63 | 0.74 | 35.56 | 34.99 | 8.53 | 5.59 |
| Yemen | 0.45 | 0.52 | 14.45 | 18.97 | 14.45 | 18.97 |
| Zambia | 0.51 | 0.59 | 46.13 | 28.42 | 16.61 | 18.68 |
| Zimbabwe | 0.47 | 0.47 | 65.75 | 21.45 | 18.58 | 21.45 |

References

1. Riebler A, Held L. Projecting the Future Burden of Cancer: Bayesian Age-Period-Cohort Analysis with Integrated Nested Laplace Approximations. *Biom J* (2017) 59(3):531-49. Epub 20170131. doi: 10.1002/bimj.201500263.

2. Kiyoshige E, Ogata S, O'Flaherty M, Capewell S, Takegami M, Iihara K, et al. Projections of Future Coronary Heart Disease and Stroke Mortality in Japan until 2040: A Bayesian Age-Period-Cohort Analysis. *Lancet Reg Health West Pac* (2023) 31:100637. Epub 20221115. doi: 10.1016/j.lanwpc.2022.100637.

3. Gupta PD. Standardization and Decomposition of Rates: A Users's Manual. *washington d* (1993).

4. Das Gupta P. Standardization and Decomposition of Rates from Cross-Classified Data. *Genus* (1994) 50(3-4):171-96.

5. Chevan A, Sutherland M. Revisiting Das Gupta: Refinement and Extension of Standardization and Decomposition. *Demography* (2009) 46(3):429-49. doi: 10.1353/dem.0.0060.

6. Thanassoulis E. Benchmarking with Dea, Sfa, and R. (2012).

7. Agasisti T, Torre EDL, Martì S. Assessing Organizations' Efficiency Adopting Complementary Perspectives: An Empirical Analysis through Data Envelopment Analysis and Multidimensional Scaling, with an Application to Higher Education. *Springer US* (2016).

8. Cooper W, Seiford L, Zhu J. Handbook on Data Envelopment Analysis. (2011).

9. Congdon P. A Spatio-Temporal Autoregressive Model for Monitoring and Predicting Covid Infection Rates. *J Geogr Syst* (2022) 24(4):583-610. Epub 20220426. doi: 10.1007/s10109-021-00366-2.

10. Lee D. A Comparison of Conditional Autoregressive Models Used in Bayesian Disease Mapping. *Spat Spatiotemporal Epidemiol* (2011) 2(2):79-89. Epub 20110312. doi: 10.1016/j.sste.2011.03.001.

11. Ren Z, Wang S, Liu X, Yin Q, Fan J. Associations between Gender Gaps in Life Expectancy, Air Pollution, and Urbanization: A Global Assessment with Bayesian Spatiotemporal Modeling. *Int J Public Health* (2023) 68:1605345. Epub 20230510. doi: 10.3389/ijph.2023.1605345.

12. Li J, Wang S, Han X, Zhang G, Zhao M, Ma L. Spatiotemporal Trends and Influence Factors of Global Diabetes Prevalence in Recent Years. *Soc Sci Med* (2020) 256:113062. Epub 20200519. doi: 10.1016/j.socscimed.2020.113062.

13. Wang S, Ren Z, Liu X, Yin Q. Spatiotemporal Trends in Life Expectancy and Impacts of Economic Growth and Air Pollution in 134 Countries: A Bayesian Modeling Study. *Soc Sci Med* (2022) 293:114660. Epub 20211218. doi: 10.1016/j.socscimed.2021.114660.

14. Bivand R, Gómez-Rubio V, Rue H. Spatial Data Analysis with R-Inla with Some Extensions. *Journal of Statistical Software* (2015) 063(20):1-31.
